# Promoting respectful maternity care using a behavioral design approach in Zambia: results from a mixed-methods evaluation

**DOI:** 10.1186/s12978-022-01447-1

**Published:** 2022-06-20

**Authors:** Jana Smith, Allison Schachter, Rachel Banay, Emily Zimmerman, Ariadna Vargas, Abigail Sellman, Ameck Kamanga

**Affiliations:** 1 ideas42, New York, USA; 2 Jhpiego, Lusaka, Zambia

**Keywords:** Respectful maternity care, Qualitative, Zambia, Disrespect and abuse, Providers, Behavioral science, Behavioral economics, Provider behavior change, Experience of care, Maternal care

## Abstract

**Background:**

Respectful maternity care (RMC) has been elevated in the global discourse, however, instances of disrespect and abuse remain prevalent. While several studies have highlighted promising approaches to promote RMC, this body of literature is still limited and few approaches have been scaled outside the initial study sites. Building on formative research conducted through a behavioral science lens, we sought to develop and test evidence-based, low-cost solutions to promote RMC which would be well-positioned for scale-up. Our study highlights the effectiveness of the solution package on provider provision of respectful care and client satisfaction, as well as intermediary outcomes and behavioral mechanisms.

**Methods:**

A quasi-experimental evaluation, informed by the behavioral design approach, was completed to test the effectiveness of a 5-component solution package in Chipata, Zambia. Quantitative surveys were collected from health facility providers and postpartum clients at baseline and endline in intervention and comparison facilities. Additional qualitative interviews were conducted with health facility providers and postpartum clients at endline. We also conducted interviews with health facility in-charges and observed labor and delivery practices at intervention facilities over the course of implementation.

**Results:**

Evidence suggested that at endline, clients at implementation facilities were less likely to experience disrespect and abuse compared to clients at comparison facilities (ß = − 0.15 p = 0.01). Clients at intervention facilities were more likely to request pain management compared to clients at comparison facilities (ß = 0.33, p = 0.003). The solutions were simple for providers to implement and were easily integrated into existing services by providers during labor and delivery. Providers at intervention facilities also described the pain management toolkit as helpful in expanding the types of pain management techniques used during labor.

**Conclusions:**

The results of this small-scale study act as a proof of concept, demonstrating that the behavioral design approach can lead to solutions that show potential for impact. In other settings where providers face similar barriers to providing RMC, an adaptation of this solution package might lead to similarly positive results. Given the global scale of disrespectful care, these low-cost solutions hold promise for improving the quality of care women receive during labor and delivery.

**Supplementary Information:**

The online version contains supplementary material available at 10.1186/s12978-022-01447-1.

## Plain Language Summary

Instances of disrespect and abuse during childbirth are prevalent around the world, particularly in low-resource settings. Few interventions have been designed and evaluated in these settings and even fewer in Southern Africa. This study aimed to understand the effects of a behaviorally informed intervention on the provision of respectful maternity care. We performed an evaluation of a health facility-based intervention, in Chipata District, Zambia. The study included quantitative and qualitative surveys with health care providers and women who recently delivered, as well as health facility observations and qualitative interviews with health facility supervisors. Our results show that clients who delivered at a facility where our intervention took place had a decreased likelihood of experiencing disrespect and abuse and an increased provision of pain management support. Our findings suggest that facility-based approaches informed by behavioral science have the potential to increase provision of respectful care and decrease the prevalence of mistreatment in low-resource settings. A large-scale evaluation of these interventions across settings could contribute valuable evidence around low-cost solutions to promote respectful maternity care.

**Supplementary Information:**

The online version contains supplementary material available at 10.1186/s12978-022-01447-1.

## Background

Respectful maternity care (RMC) has been elevated in the global discourse, however, instances of disrespect and abuse remain prevalent: a 2019 four-country (Ghana, Guinea, Myanmar and Nigeria) WHO study found that 35 percent of women surveyed had experienced “physical or verbal abuse, or stigma or discrimination” during childbirth [1]. While several studies have highlighted promising approaches to promote RMC, this body of literature around interventions is still limited compared to the evidence base which documents the prevalence of mistreatment [2–4].

Prior studied intervention packages include workshops for health workers and an antenatal care education program for patients in Tanzania, a client service charter and maternity quality improvement processes in Tanzania, and provider training and support coupled with several community-level interventions in Kenya [2–4]. Our research sought to build on and complement this work by testing interventions that shift the environment of providers during the moment of care provision, rather than prior to or after care provision. We also sought to evaluate interventions which would require minimal implementation resources and to conduct this work in southern Africa, specifically in Zambia, where previous research on RMC interventions had not been as widely conducted.

Formative research conducted through a behavioral science lens in Chipata, Zambia demonstrated that disrespectful care was prevalent and providers experienced several behavioral barriers to provision of respectful maternity care [5]. Building on these findings, we developed and tested evidence-based, low-cost solutions to promote RMC that have the potential to be scaled. This study describes an approach for developing evidence-based interventions and shares findings on the impact of this solution package on final outcomes as well as intermediary outcomes and behavioral mechanisms. The aim of this research is to further the understanding of approaches to address disrespect and abuse during labor and delivery and to improve women’s experience of care.

## Methods

### Intervention design

We employed Datta and Mullainathan’s [6] behavioral design approach to systematically design the interventions. This approach has been used to address a wide range of public health challenges in low- and middle-income countries including gender-based violence, reproductive health, and neglected tropical diseases [7–9]. First, we defined the problem and then conducted qualitative, formative research to identify the behavioral barriers inhibiting the provision of RMC. The behavioral barriers and their accompanying contextual features identified in this formative research are described in Table 1 and are published elsewhere [5].

**Table 1:** Mapping of behavioral drivers to interventions

Behavioral barrier	Drivers that underlie behavioral barriers	Pain management toolkit	Provider–client promise	Feedback box	Reflection workshop
Providers believe they are already doing everything they are supposed to do	Training, supervision and feedback is focused on clinical treatment and health risks and does not address respectful care	X		X	X
	There are clinical algorithms and guidelines, including visual cues in the facility, but nothing which provides clear guidelines for how to give good care	X	X		
	Pain is seen as a natural birth experience—the provider had a painful delivery, has attended many painful deliveries, and the bible says that labor is painful	X	X		X
	Provider has attended many deliveries and developed a “feel” for how care is provided	X			X
Harsh treatment is considered normal	Provider experienced violence as a child as a form of discipline				X
	Training and clinical experience of provider reinforces that clients need rigid, forcefully delivered commands and interventions		X		X
Providers believe they do not need to provide respectful care	There are no serious consequences to providers who engage in disrespectful or abusive behavior			X	X
	Client clothing or appearance makes them seem low-income, or they are considered to be a community member of lower status		X	X	X
	Provider has never interacted with the client before delivery and the client is behaving erratically or not following instructions	X	X		X
Providers perceive that the costs of respectful care outweigh the gains	Maternal or infant death results in an audit, placing an emphasis on clinical practices			X	X
	Providers do not receive salient information or feedback on the impact of respectful or disrespectful care on health outcomes	X			X
Providers believe that disrespectful care will assist their clinical objectives	Client does not follow provider instructions in part due to lack of rapport, extreme pain and fatigue	X	X		X

Using insights derived from the formative research, we then developed a solution package using an iterative co-design process with a range of different stakeholders including the District Health Office of Chipata, the local team from our implementing project partner (SM360+), the research team, clinicians (midwives and their supervisors), and women in labor. The co-design process resulted in the design of a solution package consisting of five components described below.

1. BETTER pain management toolkit: The toolkit was intended to incorporate pain management into routine client care. BETTER stands for Breathe, Encourage, Turn, Think, and Rub. The toolkit includes (1) Pain management technique posters to cue both providers and clients to pain management support (2) Pain management manual, (3) massage balls, and a (4) pain management partograph guide.
2. Feedback box: The feedback box was intended to empower clients to share feedback and provide the means to regularly assess clinic performance. Women were provided with a token upon discharge from the maternity ward and instructed to insert the token into the slot that best reflected the service they received.
3. Provider–client promise: The promise sought to clarify and set expectations for behavior of both providers and clients and reassure clients of the treatment they should receive. Providers promised to encourage and support the client, explain why procedures are needed, help to manage pain and not to yell, scold or slap the client. Clients promised not to push until the providers says so, to allow the provider to examine her, to lie on her side when asked and to let the provider know when she was in pain or had a question. The document was read aloud by providers upon admission to the labor ward and was signed by both provider and client.
4. Fresh start funds: The funds were intended to generate a sense of “fresh start” for the staff and a sense of agency in changing the experience of care. Facilities were provided with a small fund (5000 Kwacha, approximately USD 300), which they used to make small changes to the labor ward to improve the non-clinical client experience.
5. Reflection workshop: The workshop encouraged providers to reflect on client care, build an intention to change care as a facility, and introduce solutions.

**Fig. 1:**
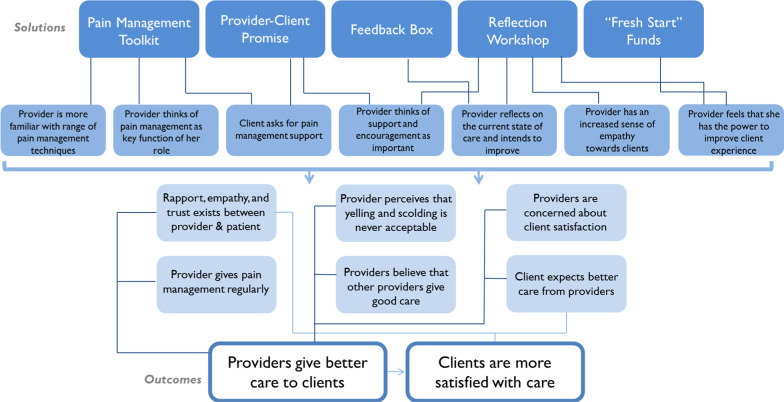
Theory of change of the intervention package

To inform our approach, we also constructed a theory of change (see Fig. 1) of our solution package drawing from our formative research and other published literature.

#### Evaluation design

To evaluate the effectiveness of the solution package, we implemented a quasi-experimental study design. The study was conducted between September and December 2019. Quantitative survey data was collected from health facility providers and postpartum clients during a baseline and endline data collection. All provider baseline quantitative surveys were administered prior to October 2019, prior to the start of the intervention. Baseline client interviews were collected through mid-October 2019 and only included postpartum clients who delivered prior to implementation. Client endline data collection began in mid-November 2019 and included women who delivered within the implementation period. Provider’s endline data was collected in December 2019. Additional qualitative data was collected at endline from both providers and clients to provide additional insight that could not be captured quantitatively. Additionally, for those at the intervention facilities, we qualitatively explored their experience with the solution set. We also conducted interviews with health facility in-charges and observed labor and delivery practices at intervention facilities.

### Study setting

Chipata District, Eastern Province, Zambia was identified as the area of implementation. As an urban area, Chipata has the largest population, and highest population density in Eastern Province [10] as well as a number of health facilities with relatively high client volumes. Chipata was also identified as a priority district for our local partner organization.

We conducted an evaluation of the solution package from September through December 2019. The evaluation was conducted in 10 urban and rural public facilities. Facilities were eligible for inclusion if our local partner, SM360+, operated programs there. The solution package was implemented in five facilities from October 2019 through December 2019. Five additional facilities were identified as comparisons and received no solution package. Each comparison facility was matched to a comparable intervention facility on the basis of the following criteria: average number of monthly deliveries, size of staff, size of catchment population, and distance from the district health office (city center). All facilities served clients of similar socio-economic status (Table 2).

**Table 2:** Demographics of intervention and comparison facilities

	Intervention	Comparison
Average size of population served	13,791	10,761
Average number of staff members that attend to pregnant women (includes nurses, midwives and clinical officers)	5	3
Average number of deliveries per month	52	31
Average distance from the District Health Office	34.6 km	33 km

### Study participants

Clients were eligible to participate if they delivered at the health facilities within 4–8 weeks of the time of survey administration, delivered a live birth and were 18 years or older [11]. All facility providers who support labor and delivery services at the facility were invited to participate in the survey. Different clients were surveyed at baseline and endline. While all facility staff who supported labor and delivery services at the health facility were invited to participate in the survey at baseline and endline, not all providers who participated in the baseline survey were available at endline. Therefore, we measured changes in outcomes at the facility level rather than at the individual level.

### Materials and processes

Data was collected by a research consultant and a member of the local partner’s monitoring and evaluation team. In Chipata, the district lead from our local partner organization facilitated introductions to staff at all ten health facilities. We obtained permission from facility supervisors to speak with and observe their staff, and conducted an informed written consent process with each participant before beginning the interview. We obtained ethical approval from a U.S.-based as well as a local Zambian Institutional Review Board. Participants received 60 kwacha (approximately $4 USD) as compensation for their time. Surveys and interviews with clients as well as facility observations were conducted by female data collector while the provider interviews were conducted by both male and female data collectors. Surveys were conducted with only one data collector present. The questionnaires used in the study were developed for the purpose of this study though validated measures were adapted and included such as Maslach Burnout Inventory section on empathy and dehumanization, Jefferson Scale of Physician Empathy and the Perceived Stress Scale. Measures for disrespect and abuse were adapted from evaluations completed by Asefa et al. [12], Population Council’s HESHIMA project [13] and the Maternal and Child Health Integrated Program Respectful Maternity Care Indicator Compendium [14].

Hour-long surveys and interviews were conducted at the facilities at times when providers were not otherwise occupied with professional obligations. Surveys with providers solicited quantitative information on provider background, professional responsibilities, typical client behaviors and providers’ responses to these behaviors, and elements of respectful care. Surveys were used to capture providers’ perspectives on patient experience, levels of rapport, empathy and trust between providers and clients and to understand providers’ perceptions of their role and disrespectful care. At endline, additional qualitative questions were added to the surveys to develop a more nuanced understanding of provider perceptions related to RMC as well as understand intervention facility providers’ experience implementing the solution package.

Safe Motherhood Action Group (SMAG) volunteers, community members who liaise between health facilities and clients in the community, helped our research team identify and make introductions to eligible clients. Eligible clients were invited to the health facility to participate in the hour-long surveys. Surveys with clients solicited quantitative information on participant background, the client birth experience, provider–client dynamics, and positive and negative elements of the birth experience. Additional qualitative questions were added to the surveys at endline to develop a more nuanced understanding of clients’ experiences and to learn more about client interaction with the solution package. Qualitative interviews with supervisors concerned participant background, supervisory responsibilities, experience implementing the solution package and provider practices, and typical client behaviors.

We conducted multi-hour observations in the labor ward of the implementation facilities only. Observations were conducted when clients arrived at the labor and delivery ward, provided that clients and providers felt comfortable with our presence and gave informed consent. Observations were captured in an objective and detailed fashion: all conversations and interactions between clients and providers and any other parties present were recorded in detailed notes, as were providers’ utilization of the solution package.

### Analysis

Our theory of change was used to develop our analytic approach to assess the effectiveness of the RMC solution package and whether or not the solution package improved client experience of care during facility-based deliveries. To assess our primary outcome, “Providers give better care to clients” we measured (1) whether the client reports they experienced any instance of disrespect and abuse (2) provider reports that colleagues believe that yelling at or scolding a patient is never acceptable. Our second primary outcome, “Clients are more satisfied with care,” was measured by (1) client rated care as very good or excellent and (2) client reports that the provider treated me [the client] well during labor and delivery.

We estimated the effect of the solution package on the outcomes of interest using an ordinary least squares (OLS) regression to measure the differences in the outcome variables at endline between intervention and comparison groups. We chose to use a linear probability model to conduct our analysis due to the interpretability of the results as compared to probit and logit models and because the range of predicted probability is not wide. All the probabilities lie within the unit interval and therefore our estimates are unbiased and consistent.

To isolate the estimated effect of the solution package, we controlled for facility-level baseline averages in all regressions, whether focusing on provider or client outcomes. In addition, we controlled for observed differences between intervention and comparison provider groups at baseline and other potential confounders. We adjusted for whether a provider was a midwife rather than a nurse or doctor, the provider’s sex, years of experience in the delivery ward, and the number of deliveries attended within the last 2 weeks. In the client outcome-focused analyses, we adjusted for facility-level baseline averages for each outcome of interest, as well as a client’s marital status, age, and parity.

Because our research design did not include random assignment and, therefore, did not allow us to control for other factors that might influence both the intervention and comparison groups, we also conducted a difference-in-difference analysis to further validate the findings. This analysis explored whether outcomes in the intervention facilities were similar to what we may have expected if the intervention had not occurred. This approach assumes that intervention and comparison facilities have similar trends over time, which we could not validate due to limited data points. In instances where the difference-in-difference results did not confirm significant OLS regression findings, we believe the OLS regressions still suggest a meaningful effect, though the size of the effect may be less certain due to other factors not captured in the OLS regression. Quantitative data was analyzed using Stata/IC version 14.0.

Qualitative data was also used to develop a more nuanced understanding of the results. In-depth interviews were conducted in Nyanja (dominant local language) and audio recorded and notes were transcribed directly into English. We employed thematic analysis, drawing from the process outlined by Braun and Clarke [15]. An initial set of codes was drawn from the theory of change described above. Data were matched to each component of the theory of change, and four members of the research team individually assessed the extent to which the evidence supported or contradicted the pathway, or whether evidence was mixed using Microsoft Excel.

## Results

A total of 220 surveys of providers and clients were completed between baseline and endline.^1^ Of those surveys, 33 providers were surveyed at baseline and 35 at endline. Of the providers surveyed at baseline, 22 were available to be surveyed at endline. 60 clients were surveyed at baseline and 92 at endline, each survey representing a unique individual. Five interviews were conducted with intervention facility in-charges and ten multi-hour observations were conducted at intervention facilities. A further breakdown of survey participants is found in Table 3.

**Table 3:** Evaluation summary

	Baseline	Endline	Monitoring
Time period	September–mid October 2019	Mid November–December 2019	Oct–December 2019
Study sites	5 comparison 5 implementation		5 implementation
Data sources			
Client	Quantitative	Quantitative and qualitative	
	Total: 60 Intervention: 28 Comparison: 32	Total: 92 Intervention: 47 Comparison: 45	Interview with in-charge (n = 5)
Provider	Quantitative	Quantitative and qualitative	
	Total: 33 Intervention: 18 Comparison: 15	Total: 35 Intervention: 22 Comparison: 13	Observations in implementation facilities (n = 10)
Measures	Client • Client reported instances of disrespect and abuse • Client reported pain management support • Satisfaction with care • Expectations for care • Post-delivery care seeking behavior		
	Provider • Perception of disrespect and abuse • Self-reported disrespectful and abusive behavior • Witness disrespectful abuse and behavior by colleague • Knowledge of pain management techniques • Importance of support and pain management during delivery • Client requests pain management support • Pain management techniques use • Perceived Stress Scale • Provider Empathy Index		

Table 4 shows the demographics of the providers and clients surveyed both at baseline and endline.

**Table 4:** Provider and client demographics at baseline and endline, by intervention and comparison sites

	Client baseline			Client endline		
	Intervention (N = 28)	Comparison (N = 32)	Full (N = 60)	Intervention (N = 47)	Comparison (N = 45)	Full (N = 92)
Age	23.8	23.2	**23.5**	24.7	24.3	24.5
Parity	2.4	2.6	**2.5**	2.4	2.3	2.4
Marital status	95% married	94% married	**95% married**	85% married	73% married	79% married
Age of most recent child delivered, in months	1.1	1.0	**1.0**	1.1	1.1	**1.1**

	Provider baseline			Provider endline		
	Intervention (N = 18)	Comparison (N = 15)	Full (N = 33)	Intervention (N = 22)	Comparison (N = 13)	Full (N = 35)
Age	36	39	38	37	36	37
Percent female	100%	67%	85%	86%	62%	77%
Midwife	56%	53%	55%	55%	54%	54%
Years of experience attending deliveries	9.4	10.1	9.7	9.9	8.2	9.3
No. delivery in past 2-weeks	3	3	3	2	2	2

There were no statistically significant differences between baseline and endline populations

We have summarized key findings below and provided additional detail on each category of outcome measure in the following text.

### Key findings summary

- Clients at implementation facilities were 15 percentage points less likely to experience any form of disrespect and abuse compared to clients at comparison facilities (ß = − 0.15 p = 0.01)
- Clients at intervention facilities were 33 percentage points more likely to request pain management compared to clients who delivered at comparison facilities (ß = 0.33, p = 0.003)
- Providers at intervention facilities reported greater use of more evidence-based pain management techniques at endline relative to baseline

### Disrespect and abuse

OLS findings suggest that at endline, clients at implementation facilities were significantly less likely, 15 percentage points, to experience any form of disrespect and abuse (including lack of privacy, threats, delivering alone and abandoned, and being made to feel uncomfortable) compared to clients at comparison facilities (ß = − 0.15 p = 0.01). The difference-in-difference analysis did not validate this finding (ß = 0.05 p = 0.61; Table 5).

**Table 5:** Client reports experiencing disrespect and abuse during labor and delivery

	Treatment		Control	
	Baseline	Endline	Baseline	Endline
Proportion of client reports that reported experiencing any instance of disrespect (%)	0.04	0.02	0.22	0.16

	OLS regression (n = 75)	Difference-in-difference (n = 152)
Intervention ß, (SE)	− 0.15** (0.06)	0.05 (0.10)
p-value	0.01	0.61

Model adjusted for: marital status, age, parity, baseline facility averages

***p < 0.01, **p < 0.05, *p < 0.1

At baseline, the percentage of providers who reported ever witnessing disrespectful care by colleagues was relatively high, with 72% of intervention providers and 81% of comparison providers reporting that they had ever witnessed disrespect and abuse by colleagues. Providers also reported witnessing all four kinds of disrespect and abuse that we explored either within the last 2 weeks or ever witnessing (use of force, threatening client, showing disrespect due to client attribute, and scolding), though scolding was the most commonly reported form. Despite high rates of witnessing disrespectful care, providers generally reported that their colleagues treated clients acceptably on a scale of totally unacceptable to perfectly acceptable.

Evidence on whether there was a change in provider’s perception that yelling or scolding is never acceptable amongst colleagues was not clear. Findings from the OLS at endline, though not statistically significant, suggest that providers at the intervention facilities were more likely to state that providers at their facility believe that yelling at or scolding a patient is never acceptable compared to providers at comparison facilities (ß = 0.5, p = 0.09; Table 6).

**Table 6:** Provider believes that yelling and scolding is never acceptable amongst their colleagues

	Treatment		Control	
	Baseline	Endline	Baseline	Endline
Proportion of provider who believe that believe that yelling or scolding a patient is never acceptable amongst their colleagues (%)	0.22	0.36	0.67	0.46

	OLS (n = 30)	Difference-in-difference (n = 68)
Intervention ß, (SE)	0.50* (0.28)	0.30 (0.24)
p-value	0.09	0.21

Model adjusted for: cadre, gender, years of experience attending deliveries, number of deliveries within the last 2 weeks and baseline facility averages

***p < 0.01, **p < 0.05, *p < 0.1

Qualitatively a few providers remarked that if a client ‘broke’ the provider–client promise, then they were justified in scolding, therefore this result is not clear. The difference-in-difference analysis did not confirm the regression results (ß = 0.30, p = 0.21).

We also adapted the Maslach Burnout Inventory section on empathy and dehumanization,^2^ to measure provider burnout and decipher whether it was linked to disrespectful care. Survey results found low levels of burnout at baseline across providers (mean values of 5 on a scale of 0–42). We also found no correlation between provider burnout and self-reported instances of disrespectful care.

### Provision of pain management

As per our theory of change, pain management support was an important intermediary to provision of better care. To identify whether the intervention impacted provision of pain management we considered several outcomes including frequency of use of pain management techniques as reported by providers, whether provider considers pain management support as one of the three most important tasks completed during labor and delivery, and whether the client reports requesting pain management support.

Clients were asked whether they requested help from the provider when they were feeling pain during labor and delivery. OLS results show that at endline, clients at intervention facilities were 33 percentage points more likely to request pain management compared to clients who delivered at comparison facilities (ß = 0.33, p = 0.003). 70% of clients at intervention facilities requested support compared to 36% of clients at comparison facilities at endline. The difference-in-difference analysis confirmed these results (ß = 0.33, p = 0.04; Table 7).

**Table 7:** Client requests pain management support

	Treatment		Control	
	Baseline	Endline	Baseline	Endline
Proportion of clients that requested pain management support (%)	0.61	0.7	0.59	0.36

	OLS regression (n = 75)	Difference-in-difference (n = 152)
Intervention ß, (SE)	0.34*** (0.11)	0.34** (0.16)
p-value	0.003	0.04

Model adjusted for: marital status, age, parity, baseline facility averages

***p < 0.01, **p < 0.05, *p < 0.1

We also asked providers to select the three most important tasks they do during delivery from a pre-determined list of common tasks observed in our formative research. At endline, though not statistically significant, OLS findings suggest that providers at intervention facilities were 29 percentage points more likely to rate pain management as one of the most important tasks during delivery compared to providers at comparison facilities (ß = 0.29, p = 0.06). Moreover, 23% of providers at intervention facilities selected pain management as important, compared to 8% of comparison providers at endline. However, the difference-in-difference analysis did not confirm the results (ß = 0.16, p = 0.37) (see Additional file 1: Table S1). Nonetheless, qualitative findings suggest that the intervention had a meaningful effect. For instance, providers described how the solutions helped emphasize their responsibility to provide pain management. They also described how the provider–client promise served as another reminder of the importance of providing pain management.

Survey data did not indicate a significant change in the number of pain management techniques a provider could recall or used between intervention and comparison groups (see Additional file 1: Table S2), but there was a positive trend in the use of more effective and technical pain management techniques amongst intervention providers. At baseline the most commonly cited techniques used by intervention providers when a client requested pain management were massage, encouragement and chat. At endline, the three most commonly applied techniques were massage, breathing exercises and change position, which were all techniques outlined in the BETTER pain management toolkit. Qualitatively, providers at intervention facilities also described the pain management toolkit as playing a role in expanding the types of pain management techniques used during labor:

*“Before the orientation I would just tell the client to do breathing exercises that when she does breathing exercises and has enough oxygen, the pain will reduce, but after orientation if the client can’t manage to do breathing exercises and has back pain I can use the ball to rub her back. So now we have a number of pain management techniques we are using to relieve the clients’ pain.”*

Moreover, several clients reported the massage ball as something that they particularly enjoyed and something different from previous deliveries. One client from an intervention facility noted, *“I loved the way they treated me and the use of a ball to rub my back, the way they used to talk to me when in pain, and the way they encouraged me.”—Intervention Facility Client.*

### Agency to improve quality of care

The section below describes outcomes related to provider’s agency to improve quality of care. As described in the theory of change, a mechanism for provision of better care was that “Provider reflects on the current state of care and intends to improve.” To measure providers’ agency, we measured providers’ interest in improving care as well as perceived need for improvement.

Most providers, in both intervention and comparison facilities, reported that they were very or extremely interested in improving care at facilities at baseline; on a scale of 1–5, 5 being extremely interested, the average response at baseline across providers was 4.5. Despite expressing interest in improving care, most providers at intervention and comparison facilities did not report a need to improve. When asked to describe the state of care of their facility, ranging from “the facility provides excellent care with little to improve” to “the facility does not provide good care and could improve in many areas,” most providers across intervention and comparison facilities evaluated the state of care favorably with “facility provides good care with a few areas to improve.” Providers, in both intervention and comparison facilities, generally reported feeling able to improve client experience during delivery with no significant differences between intervention and comparison at endline.

### Rapport, empathy, and trust between provider and patient

The section below describes outcomes related to the intermediate outcome described in the theory of change, “Rapport, empathy and trust exists between provider and patient”. Questions were asked of both providers and clients to measure this outcome including an index to measure provider empathy, whether a client reports trusting their provider, report that the provider cared for them and clients’ belief that their satisfaction was important to the provider.

Empathy was measured through an index, based on responses to six different questions, including whether a provider reported “understand[ing] what is going on in my clients’ minds by paying attention to their nonverbal cues and body language” or agreed that “my clients feel better when I understand their feelings,” statements adapted from the Jefferson Scale of Physician Empathy [16]. At endline, as measured on a scale of 0–5, though not statistically significant, findings suggested that providers in intervention facilities were more likely to be more empathetic towards clients (ß = 0.20, p = 0.07) as compared to providers at comparison facilities. The results were, however, not corroborated by the difference-in-difference analysis (ß = 0.03, p = 0.83) (see Additional file 1: Table S3).

At both baseline and endline, almost all clients reported trusting their provider, feeling that their provider cared for them, and believing that their satisfaction was important to providers. However, clients’ qualitative reflections were mixed. Several clients described feeling a sense of relief at being promised the kind of care described in the provider–client promise, indicating that this kind of care was not necessarily what they had expected. Clients reported that they felt confident that the provider would follow their promise, and none reported feeling that the promise had been broken during her delivery. Clients also remarked that the promise was educational and that they valued being consulted and involved.

### Client expectations and satisfaction

Below we describe findings from our primary outcome of interest, “Clients are more satisfied with care” as well as expectations of care, which was measured across several different aspects of care described below.

While clients reported being satisfied with care overall, clients’ expectations for respectful care were low and did not increase during implementation. At baseline, across intervention and comparison facilities, almost half of clients said they expected that a provider would yell or scold them and a third said they expected the provider might use insults, intimidations, threats, or coercion. Several women explicitly mentioned that they expected to be shouted at or slapped either because they were arriving late to the facility or because this is what they had heard from others. These values remained high at endline; 40% of intervention and comparison clients expected their provider to yell or scold them during labor and delivery and 32% expected their provider might use insults, intimidations, threats or coercion.

Despite having an expectation of disrespectful care, almost all clients, across intervention and comparison facilities, also reported an expectation that providers would provide “good care” at baseline and values remained high at endline. Not being shouted at or beaten or having the provider’s assistance with anything not immediately essential to a safe delivery (such as helping to clean up bodily fluids after delivery) were described as reasons to be particularly satisfied with the care received rather than examples of care one should expect.

Across intervention and comparison facilities, the endline qualitative findings suggest that women’s low expectations of provider–client interpersonal care, may be linked to their focus on the baby’s survival. Several women explained that they perceived a real risk that the baby might not survive, and allowed themselves to develop attachment to the baby only once they felt certain that the baby would live. Clients also mentioned that their primary concern during delivery was delivering a healthy baby, and our qualitative data suggested that even when clients expected disrespect and abuse, they reported being satisfied by the care they received if they delivered a healthy baby.

While our study did not detect a significant impact of the intervention on quantitative measures of client satisfaction or the importance of client satisfaction to providers, qualitative results suggest that both clients and providers at intervention facilities found utility in the feedback box. Providers described the feedback box as a means to understand client satisfaction;

“*For example, we are having unsatisfied clients, it will help us look into the matter and see where we are having the problem. If the clients are very satisfied and we have a lot of tokens then we know that we are doing our job and clients are appreciating if they are satisfied because of one or two things that they are not happy about, we try to talk among ourselves and try to solve the issue so that all the mothers can go home happy.”*—Intervention Facility Provider.

Clients from intervention facilities described “feeling good” about being asked to share their level of satisfaction through the feedback box. Additionally, clients commonly noted that they believed that positive feedback would be motivational for providers and that negative feedback would lead providers to change, thus suggesting their confidence in the feedback mechanism.

## Discussion

This evaluation provides promising evidence of the potential for behaviorally-informed solutions to increase provision of RMC and the use of behavioral design to develop effective interventions. Our participatory design led to interventions which were feasible to implement without a significant investment of resources and were well-received by clients and providers. While we were unable to attribute the observed effects to particular components of the solution package, our results suggest that all but one of the five solutions, the fresh start funds, contributed meaningfully to these positive results. This aligns with the findings from our formative research phase, and highlights the value of the behavioral design approach which allowed us to identify a range of behavioral drivers impacting provision of RMC and established the need for a multi-faceted solution to address these challenges [5]. Our results also generally align with those of previous research on RMC interventions, particularly those of the *Staha* project given their use of a comparison group in their study design, though we employed slightly different measures to capture instances of disrespect and abuse [2]. Other RMC intervention studies did not capture some of the other intermediate outcomes and behavioral mechanisms we measured and therefore those results cannot be compared.

One specific finding from our formative research was that providers had a narrowly defined mental model of their role which focused on clinical tasks and avoiding client or infant death [5]. One objective of the solutions was to expand the providers’ mental model of their role to include pain management support as a means for improving client experience. The approach we employed was multi-faceted (role play with techniques, a manual, a reminder poster by the client bedside, a partograph guide by the provider’s desk, and a massage ball) as research suggests that mental models are formed through repeated and continuous interaction [17]. Through our design process, we were able to identify a range of contextualized touchpoints which could give providers repeated interaction with pain management cues. Our evaluation findings suggest that the interventions shifted provider perception of their role, as there was a significant increase in providers citing pain management as a priority task, and also that this mechanism contributed to improved pain management provision. These findings point to strengths in the behavioral design approach, both in identifying the appropriate behavioral mechanisms to target as well as choosing effective intervention points to activate these mechanisms. While our solutions were limited to those which could be deployed at the facility level, additional opportunities exist to reshape providers’ mental model of their role through the addition of pain management content in midwifery school curriculum.

Client satisfaction was a key outcome of interest in our evaluation as were other intermediate outcomes related to client expectations of care. The challenges in accurately capturing client satisfaction are well-established in the literature [18, 19], and our results suggest that measures other than client satisfaction may be more appropriate for identifying opportunities for improvement which are aligned with client desires. Our evaluation findings, which align with findings from our formative research, suggest that reported satisfaction was linked to whether the highest priority of the client was met, rather than whether or not the experience was satisfactory across a range of components of interest. That is, clients’ greatest concern was delivering a healthy baby and as long as that expectation was met, they also reported high levels of satisfaction and assessed “good care” and “trust” through this lens. Measurements of client satisfaction intend to capture opportunities for improvement, but since clients, in all service interactions, will assess satisfaction as related to expectations and pressing priorities, satisfaction as a means for assessing the match between desired experience and actual experience falls short. Measures should instead capture a range of different features clients deem important to their care experience since otherwise, one particularly weighty element (the infant’s safety) may skew reported satisfaction and obscure opportunities for making care more respectful and responsive to client desires. Clear, specific measures of dimensions of client satisfaction may also facilitate providers’ recognition of areas for improvement, since our findings also suggest that most providers are interested in improving care without feeling a strong reported need for improvement.

Our evaluation has a few potential limitations. First, it was not feasible to randomly assign facilities to intervention or comparison conditions, which makes it more challenging to distinguish between effects driven by the intervention and those due to differences between the intervention and comparison facilities and providers for which we were unable to adjust. Our regression analyses adjusted for differences that we were able to observe, and we conducted a difference-in-difference analysis to substantiate findings from our regression analysis, but it remains possible that unobserved differences in characteristics and trends between the two groups influenced our results and may also have contributed to instances where the OLS and difference-in-difference findings did not align. This may also suggest that the size of the effect found from the OLS regressions may be due in part to factors for which we were unable to control.

We used insights from qualitative interviews as appropriate to strengthen the analysis. Another limitation is that we were only able to conduct the evaluation in a small number of facilities for a short duration of just over 2 months and with a small sample. The short duration of the evaluation may not fully capture the impact of the interventions implemented, and the small sample means there was less power to detect differences. Possibly because of the limited size of the sample, the difference-in-difference results did not always validate the OLS findings.

Another limitation of our findings relates to challenges in implementation of the interventions, particularly the reflection workshop. Provider participants changed between baseline and endline data collection rounds, and while materials were provided to orient providers to the solution package, it is not clear that providers who did not attend the workshop received the same intensity of the intervention which may also impact results and minimize observable impact of the intervention.

## Conclusions

The results of this study demonstrate the potential impact of the solution package we developed and implemented to address drivers of disrespectful and abusive care during labor and delivery in Zambia. These results support the case for a larger scale evaluation to further validate the effectiveness of the solutions and identify the relative effects of the different components of the solution package. In other settings where providers face barriers to providing RMC similar to those identified in our formative research, an adaptation of this solution package might lead to similarly positive results. Given the global scale of the problem of disrespectful care, these low-cost solutions hold promise for improving the quality of care women receive during labor and delivery.

## Supplementary Information

**Additional file 1: Table S1.** Provider believes that pain management is one of the three most important tasks during labor and delivery. **Table S2.** Number of pain management techniques provider can recall. **Table S3.** Provider Empathy Index on a scale of 0 (low) to 5 (high).

**Additional file 2.** Data collection instruments.

## Data Availability

The datasets used during the current study are available from the corresponding author on reasonable request.

## Abbreviations

OLS: Ordinary least squares regression
RMC: Respectful maternity care
SM360+: Safe Motherhood 360+
SMAG: Safe Motherhood Action Group

## Footnotes

1 We did not track the number of individuals who refused to participate.

2 The Maslach Burnout Inventory is typically used to self-assess level of provider burnout. A subset of the questions was modified and adapted for use in our survey, specifically to measure burnout as it relates to dehumanization and empathy.
