# Supplementary material for: Promoting respectful maternity care using a behavioral design approach in Zambia: results from a mixed-methods evaluation
Source: Reprod Health. 2022 Jun 20;19:141. doi: 10.1186/s12978-022-01447-1 (PMC9208205; doi:10.1186/s12978-022-01447-1)
Supplement: Supplementary file 1 — Additional file 1: Table S1. Provider believes that pain management is one of the three most important tasks during labor and delivery. Table S2. Number of pain management techniques provider can recall. Table S3. Provider Empathy Index on a scale of 0 (low) to 5 (high). [file 12978_2022_1447_MOESM1_ESM.pdf]

Supplmental Table 1: Provider believes that pain management is one of the three most important tasks during labor and delivery

	Treatment		Control	
	**Baseline**	**Endline**	**Baseline**	**Endline**
Proportion of provider that stated pain management is one of the most important things during labor and delivery (%)	0.11	0.23	0.13	0.08

	OLS Regression	Difference-in-difference
	(n=75)	(n=152)
Intervention ß, (SE)	0.29*, (0.15)	0.16, (0.18)
p-value	0.06	0.37
Model adjusted for: marital status, age, parity, baseline facility averages		
**** p<0.01, ** p<0.05, * p<0.1*		

Supplemental Table 2: Number of pain management techniques provider can recall

	Treatment		Control	
	**Baseline**	**Endline**	**Baseline**	**Endline**
Number of pain management techniques provider was able to recall	2.7	3.18	1.93	2.46

	OLS	Difference-in-difference
	(n=30)	(n=68)
Intervention ß, (SE)	0.19, (0.47)	-0.09, (0.53)
p-value	0.75	0.87
Model adjusted for: cadre, gender, years of experience attending deliveries, number of		
	**** p<0.01, ** p<0.05, * p<0.1*	

Supplemental Table 3: Provider Empathy Index on a scale of 0 (low) to 5 (high)

	Treatment			
	**Baseline**	**Endline**	**Baseline**	**Endline**
Empathy Scale Index on a scale of 0 (low) to 5 (high)	4.03	3.96	4.01	3.91

	OLS	Difference-in-difference
	(n=30)	(n=68)
Intervention ß, (SE)	0.20*, (0.10)	0.03, (0.14)
p-value	0.07	0.83
Model adjusted for: cadre, gender, years of experience attending deliveries, number of		
**** p<0.01, ** p<0.05, * p<0.1*		
