# Supplementary material for: Promoting respectful maternity care using a behavioral design approach in Zambia: results from a mixed-methods evaluation
Source: Reprod Health. 2022 Jun 20;19:141. doi: 10.1186/s12978-022-01447-1 (PMC9208205; doi:10.1186/s12978-022-01447-1)
Supplement: Supplementary file 2 — Additional file 2. Data collection instruments. [file 12978_2022_1447_MOESM2_ESM.pdf]

Table of Contents

Baseline Client Survey Instrument 2

Baseline Provider Survey Instrument 8

Health Facility In-Charge Survey Instrument 15

Client Endline Survey Instrument 18

Endline Provider Survey Instrument 26

Observation guide for Pain Management Toolkit (BETTER Poster, BETTER Pain Management Technique Manual, and BETTER Partograph Guide) 37

Observation guide for Feedback Box 40

Observation guide for Provider-Client Promise 42

Health Facility Name ________________
Health Facility ID _ _
Date __ __/ __ __/ __ __ **(DD/MM/YY)**
Pre/Post Survey _____________ **(1= Pre, 2=Post)**

Baseline Client Survey Instrument

Section I: Provision of care

Today I'd like to talk to you about the care you received during your recent delivery at the health facility.
To start, let me first learn a little about you.

Question	Answer choices	Data Entry
1. How old are you?	__ __ years	
2. How many children do you have?	*Select one*<br>__ 1<br>__ 2<br>__ 3<br>__ 4+	 **1**<br>**2**<br>**3**<br>**4**
3. What is your marital status?	*Select one*<br>__ Never Married<br>__ Living together<br>__ Married<br>__ Divorced/ Separated<br>__ Widowed	**1**<br>**2**<br>**3**<br>**4**<br>**5**
4. At what facility did you deliver your youngest baby?	__ Kasenengwa Zonal RHC<br>__ Kamlaza RHC<br>__ Mkanda Zonal RHC<br>__ Chinunda RHC<br>__ Chiparamba Zonal RHC<br>__ Madzimawe<br>__ Kapata Zonal RHC<br>__ Namseche RHC<br>__ Chikando Zonal RHC<br>__ Champhande RHC	**1**<br>**2**<br>**3**<br>**4**<br>**5**<br>**6**<br>**7**<br>**8**<br>**9**<br>**10**
5. How old is your youngest baby?	________ months	
6. Who was the main person that delivered your baby?	*Read aloud and select one response*<br>__ Doctor<br>__ Nurse<br>__ Midwife<br>__ Other __________<br>__ Do not know	 **1**<br>**2**<br>**3**<br>**4**<br>**5**
7. Do you know the name of the provider?	__ YES<br>__ NO<br><br>If no, skip to question 8	**1**<br>**0**
7a. If so, can you please provide the name		

8. How did you feel about the way the provider(s) treated you during labor and delivery?	*Read prompt aloud and select one*<br><br>__ Very satisfied<br>__ Somewhat satisfied<br>__ Somewhat dissatisfied<br>__ Very dissatisfied	 <br>**4**<br>**3**<br>**2**<br>**1**
9. Which of the following words describes the provider who attended to you?<br><br>9a.<br>9b.<br>9c.<br>9d.<br>9e.<br>9f.<br>9g.	*Read all responses below and select all that apply*<br><br><br>__ Kind/Friendly<br>__ Skilled<br>__ Patient<br>__ Rude/Harsh<br>__ Impatient<br>__ Collaborative<br>__ Violent	 **YES=1**<br>**NO=0**

Each of the questions below relate to your expectations prior to going to the facility for your most recent delivery.

Please respond whether you agree or disagree with each statement

10. I expected my provider to provide good care	__ Agree<br>__ Disagree	**1**<br>**0**
11. I expected my provider to yell or scold at me during labor and delivery	__ Agree<br>__ Disagree	**1**<br>**0**
12. I expected to have privacy during labor or delivery	__ Agree<br>__ Disagree	**1**<br>**0**
13. I expected that the provider might use insults, intimidations, threats or coercion	__ Agree<br>__ Disagree	**1**<br>**0**
14. I expected that I would have a safe delivery	__ Agree<br>__ Disagree	**1**<br>**0**
15. I expected my provider to help me manage my pain	__ Agree<br>__ Disagree	**1**<br>**0**

16. At any point during your delivery did the provider seem:<br><br>16a.<br>16b.<br>16c<br>16d.	*Read prompt aloud and select all that apply*<br><br>__ Angry<br>__ Stressed<br>__ Nervous<br>__ Disgusted	**YES=1**<br>**NO=O**
17. How painful was your delivery?	*Read responses aloud and select one*<br>__ Unbearable pain<br>__ Severe pain<br>__ Moderate pain	 **1**<br>**2**<br>**3**

	__ Slight pain<br>__ No pain	**4**<br>**5**
18. Did you ask the provider to help you when you were feeling pain?	__ YES<br>__ NO	**1**<br>**0**
19. Did the provider do anything to make you feel better or alleviate the pain?	__ YES<br>__ NO<br>If no, skip to question 21	**1**<br>**0**
20. If yes, what did they do?	*Read options aloud and select all that apply*	**YES=1**<br>**NO=O**
20a.	__ Breathing exercises	
20b.	__ Helped you think distracting or positive thoughts	
20c.	__ Encouraged you	
20d.	__ Helped you change position/walk around	
20e.	__ Rubbed/massaged you with hand or ball	
20f.	__ Chatted with you	
20g.	__ Gave medication	
20h.	__ Other<br>Please specify: __________________	
21. How important do you think your satisfaction was to the provider?	*Read responses aloud then select one*<br><br>__ Not important<br>__ Of minimal importance<br>__ Important<br>__ Very important	 <br>**1**<br>**2**<br>**3**<br>**4**

Each of the statements below relate to your experience during labor and delivery during your most recent delivery.

Please respond whether you agree or disagree

22. My provider treated me well during my delivery	__ Agree<br>__ Disagree	**1**<br>**0**
23. I trusted my provider to manage my delivery	__ Agree<br>__ Disagree	**1**<br>**0**
24. My provider cared about me and how I was feeling.	__ Agree<br>__ Disagree	**1**<br>**0**
25. I accepted that my provider might need to yell at me or scold me during labor and delivery	__ Agree<br>__ Disagree	**1**<br>**0**
26. My provider was available and listened to me during labor and delivery	__ Agree<br>__ Disagree	**1**<br>**0**

27. Did you experience any of the following during childbirth when at the facility?	*Read each prompt aloud and have client select all that apply*	*Enter*<br>*1=YES*<br>*0= NO*
27a.	__ Provider made you feel uncomfortable	

27b.	__ Provider shouted/scolded or said something harsh or unkind	
27c.	__ Provider threatened to withhold treatment	
27d.	__ Provider made threatening, negative or discouraging comments	
27e.	__ Your body was seen by others, or your privacy Was violated in another way	
27f.	__ Provider ignored or abandoned you when in need	
27g.	__ You delivered alone	
27h.	__ You had a surgery done without consenting to it	
27i.	__ Provider hit, slapped, pushed, pinched, or otherwise beat you	
28. If you were dissatisfied with the care you received, would you feel comfortable making a complaint?	__ YES<br>__ NO	**1**<br>**0**
29. How would you file a complaint, if you decided to?	*Read response aloud and select best response*<br><br>__ Notify facility staff<br>__ Tell my provider directly<br>__ Tell the hospital in-charge<br>__ Other, please specify ________________	 <br>**1**<br>**2**<br>**3**<br>**4**

Section II: Satisfaction with Service

30. Which factors did you consider when choosing this facility for your last delivery?	*Read each prompt aloud and select all that apply.*	*Enter*<br>*1=YES*<br>*0= NO*
30a.	__ Closest facility	
30b.	__ Facility provides good clinical care	
30c.	__ Providers treat clients well	
30d.	__ Same facility used for ANC	
30e.	__ To get services not available elsewhere	
30f.	__ Affordable services	
30g.	__ Recommended by relative/friend	
30h.	__ Avoid congestion in other facilities	
30i.	__ Referred from another facility	
30j.	__ Only option	
30k.	__ Facility offers specialized services	
30l.	__ Decision made by spouse	
30m.	__ Other<br>Please Specify ____________	
31. How would you rate the care you received for this delivery?	*Read response options aloud and select one response*<br>__ Excellent<br>__ Very good<br>__ Good<br>__ Fair	 <br>**5**<br>**4**<br>**3**<br>**2**

	___ Poor	**1**
32. Would you recommend this facility to a friend or relative for delivery?	___ YES<br>___ NO<br>___ UNDECIDED	**2**<br>**1**<br>**0**

33. How would you rate the importance of the following in deciding where to have a future delivery?

The response options are not at all important, slightly important, neutral, moderately important or extremely important

	Not at all important (1)	Slightly Important (2)	Neutral (3)	Moderately Important (4)	Extremely Important (5)
33a. Location of the health facility					
33b. The clinical knowledge of the provider					
33c. How the provider treats you					

Section III: Other and Future health services

34. Have you returned to any health facility since giving birth?	___Yes<br>___No<br>**If yes, continue to question 35**<br>**If no, skip to question 39**	**Yes=1**<br>**No=0**
If yes, please tell me which of these services you returned for and whether you returned to this facility or another one.<br><br>*Read each service aloud*		**1= Yes**<br>**0= No**
35a	___ Postnatal check up	**1**<br>**0**
35b.	___ Returned to this facility<br>___ Returned to other facility	**1**<br>**0**
36a.	___ Immunizations	**1**<br>**0**
36b.	___ Returned to this facility<br>___ Returned to other facility	**1**<br>**0**
37a.	___ Family planning	**1**<br>**0**
37b.	___ Returned to this facility<br>___ Returned to other facility	**1**<br>**0**
38.a	___ Other reason<br>Please Specify ______________________	**1**<br>**0**
38b.	___ Returned to this facility<br>___ Returned to other facility<br><br>**Skip to question 40**	**1**<br>**0**

39. If no, what are your reasons for not returning to the health facility	*Do not read out loud, instead listen and note the appropriate response*	
	__ Have not sought health services	**1**
	__ Did not like the care I received during my last visit	**2**
	__ Received care/services through community health workers	**3**
	__ Other<br>*Please specify* ______________	**4**

40. Would you return to the same facility for a future delivery?	__ YES<br>__ NO<br>__ UNDECIDED	**2**<br>**1**<br>**0**
41. Why would or would you not return to the facility for a future delivery?	*Do no read prompts, but select all responses related to client answers*	*Enter*<br>*1=YES*<br>*0= NO*
41a.	__ Location of facility	
41b.	__ Quality of clinical care	
41c.	__ How Providers treat clients	
41d.	__ Services available	
41e.	__ Cost of services	
41f.	__ Only option	
41g	__ Other<br>Please Specify____________________	
42. The next several questions are hypothetical. If a friend or relative was considering delivery at the health facility, what would you tell them to expect?<br><br>Please state if you agree or disagree with what your friend or relative should expect.		**Agree=1**<br>**Disagree =0**
42a. They should expect the provider to welcome them	__ Agree<br>__ Disagree	
42b. They should expect that the provider might use physical force, such as slapping or hitting	__ Agree<br>__ Disagree	
42c. They should expect that a provider might use insults or threats	__ Agree<br>__ Disagree	
42d. They should not expect to have privacy during labor and delivery	__ Agree<br>__ Disagree	
42e. They should expect that the provider may yell or be harsh with them	__ Agree<br>__ Disagree	

Health Facility Name ________________
Health Facility ID _ _
Date __ __/ __ __/ __ __ **(DD/MM/YY)**
Pre/Post Survey _____________ **(1= Pre, 2=Post)**

Baseline Provider Survey Instrument

Section 1: Provider Role

To start, I'd like to learn more about your role at the health facility as it relates to labor and delivery.

Question	Answer choices	Data Entry
1. How old are you?	|__| __|years	
2. What is your gender?	__ Male<br>__ Female	**0**<br>**1**
3. What is your title?	__ Nurse<br>__ Midwife<br>__ Doctor<br>__ Other, please specify<br>________________	**1**<br>**2**<br>**3**<br>**4**
**4.** How many years of experience do you have attending deliveries?	|__| __| years	
5. How many deliveries did you attend over the last two weeks?	*Select one*<br>__ 0<br>__ 1-4<br>__ 5-9<br>__ 10-14<br>__ 15-19<br>__ 20-29<br>__ 30-39<br>__ ≥40	 **1**<br>**2**<br>**3**<br>**4**<br>**5**<br>**6**<br>**7**<br>**8**
6. What are the three most important things a provider does during delivery?<br><br>I will read the choices below and please select 3.<br><br>*Read responses below and then note the three most important below under 6a-6c.*<br>*For data entry enter the corresponding number in the data entry fields for the three response*		
	Deliver a healthy baby	**1**
	Encourage and support the client during delivery	**2**
	Handle any complications that arise	**3**
	Ensure proper documentation	**4**
	Monitor the delivery	**5**
	Help the client to manage pain	**6**
	Ensure the client follows instructions	**7**
	Other	**8**
	Please specify : _______________________	
6a.<br>6b.<br>6c.	Response 1 __<br>Response 2 __<br>Response 3 __	

7. Which of the following statements do you most agree with?<br><br>I will read the three responses below, and then please select the one you agree with most.<br><br>*Read the prompts below, select one response.*		
	The thing that matters most is that the baby and mother are healthy	**1**
	It matters that the baby and mother are healthy and the mother is satisfied with the care she received	**2**
	It only matters that the mother is satisfied with the care she received.	**3**

8. How important is it to you to provide support and encouragement during labor and delivery?	*Read prompts aloud and select one response*<br><br>__ Not at all important<br>__ Slightly Important<br>__ Moderately Important<br>__ Very Important<br>__ Extremely Important	 <br>**1**<br>**2**<br>**3**<br>**4**<br>**5**
9. If a client is experiencing pain during labor, is there something that you do to help her manage the pain?	__ YES<br>__ NO	**1**<br>**0**
9a. What techniques can be used to manage pain?<br><br>9a1.<br>9a2.<br>9a3.<br>9a4.<br>9a5.<br>9a6.<br>9a7.<br>9a8.<br>9a9.	*Do not read. Mark the corresponding answer, and select all that apply*<br><br>__ Breathing exercises<br>__ Encourage with positive or distracting thoughts<br>__ Encouragement<br>__ Change position/walk around<br>__ Rub/massage with hand or ball<br>__ Chat with her<br>__ Gave medication<br>__ Nothing<br>__ Other<br>*Please explain-*_________________	**1= Yes**<br>**0=No**
9b. How frequently do you use the techniques?	*Read all responses aloud and select one response*<br><br>__ During every delivery<br>__ During most deliveries<br>__ During some deliveries<br>__ Rarely<br>__ Never	 <br>**5**<br>**4**<br>**3**<br>**2**<br>**1**
9c. In the last 30 days, has a client requested pain management support during labor and delivery?	__ YES<br>__ NO<br><br>If no, proceed to question 10	**1**<br>**0**

9d. If yes, did you provide pain management support?	__YES<br>__ NO	**1**<br>**0**
9e. What techniques did you use?<br><br>9e1.<br>9e2.<br>9e3.<br>9e4.<br>9e5.<br>9e6.<br>9e7.<br>9e8.<br>9e9.	*Do not read. Mark the corresponding answer, and select all that apply*<br><br>__ Breathing exercises<br>__ Encourage with positive or distracting thoughts<br>__ Encouragement<br>__ Change position/walk around<br>__ Rub/massage with hand or ball<br>__ Chat with her<br>__ Gave medication<br>__ Nothing<br>__ Other<br>*Please explain-*_________________	**1= Yes**<br>**0=No**

Section II: Provision of Care

10. Please indicate your level of agreement with the following prompts.
The choices are strongly disagree, disagree, neither agree or disagree, agree, and strongly agree

Read each prompt aloud.

	Strongly Disagree (1)	Disagree (2)	Neither Agree or Disagree (3)	Agree (4)	Strongly Agree (5)
10a. When giving birth, clients are often cooperative					
10b. Clients who have a low level of education are less likely than other clients to follow instructions					
10c. It is sometimes necessary to scold a client during labor and delivery					
10d. Clients often do not want to listen to providers during labor and delivery					
10e. Yelling at a client during labor and delivery ensures that she cooperates during procedures					
10f. Clients in labor or in delivery are deserving of kindness					

11. Have you witnessed the following actions in the labor and delivery ward?

For each action you will be asked whether you have ever witnessed the action, if you have witnessed the action in the last 2 weeks, or you have never witnessed the action			
	Never (0)	Yes, within the past 2 weeks (1)	Yes, ever in the past (2)
11a. Service providers use physical force with laboring clients (such as slapping or hitting)			
11b. Service providers use insults, intimidation, threats or coercion towards clients or their companions			
11c. Service providers show disrespect to clients based on any specific attribute (such as marital status, HIV status, economic status, age)			
11d. Service providers scold a client for her behavior or yell at her			

12. In your own personal capacity have you ever done anything that you consider to be disrespect or abuse toward a client in childbirth?	__ YES<br>__ NO	**1=YES**<br>**0=NO**
13. Please evaluate this statement: Providers in my facility believe that yelling and scolding a patient is:	*Read responses aloud and select one response*<br><br>__ Is never acceptable<br>__ Isn't acceptable but sometimes is necessary<br>__ Is acceptable when it is necessary<br>__ Always acceptable	 <br>**1**<br>**2**<br><br>**3**<br>**4**

14. The next questions ask you about your feelings and thoughts during the last month. In each case, you will be asked to indicate how often you felt or thought a certain way.

The choices are never, almost never, sometimes, fairly often, and very often

	**Never (0)**	**Almost Never (1)**	**Sometimes (2)**	**Fairly Often (3)**	**Very Often (4)**
14a. In the last month, how often have you felt that you were unable to control outcomes for clients in the ward?					
14b. In the last month, how often have you felt nervous and "stressed" when attending to a delivery?					
14c. In the last month, how often have you felt confident about your ability to handle problems in the delivery ward?					
14d. In the last month, how often have you been able to control irritations in your life?					
14e. In the last month, how often have you been angered about something that					

happened during a delivery because of things that were outside of your control?					

15. Please indicate your level of agreement with the following prompts.

The choices are strongly disagree, disagree, neither agree or disagree, agree, and strongly agree

	Strongly disagree (1)	Disagree (2)	Neither agree or disagree (3)	Agree (4)	Strongly agree (5)
15a. I try to imagine myself in my clients' life when providing care to them					
15b. I try to understand what is going on in my clients' minds by paying attention to their nonverbal cues and body language					
15c. I believe that empathy is an important therapeutic factor in medical treatment					
15d. My clients feel better when I understand their feelings					
15e. I try not to pay attention to my clients' emotions in interviewing and history taking					
15f. It is difficult for me to view things from my clients' perspectives					

16. Please indicate your level of agreement with the following prompts.

The choices are strongly disagree, disagree, neither agree or disagree, agree, and strongly agree

	Strongly Disagree (1)	Disagree (2)	Neither agree or disagree (3)	Agree (4)	Strongly Agree (5)
16a. I prefer to wash my hands pretty soon after shaking someone's hand					
16b. It does not make me anxious to be around sick people					
16c. My hands do not feel dirty after touching money					
16d. The more educated a person is, the better their hygiene is likely to be					
16e. I feel ill or disgusted when I see blood or other bodily fluids on my clothing or hands					
16f. It is wrong that unmarried girls have sex and get pregnant					

17. For each question, please tell me how often you may experience the feelings described.

The choices are never, a few times per year, once a month, a few times per month, once a week, a few times per week, and every day

	Never	A Few Times per		A Few Times per	Once a	A Few Times per	

	(0)	Year (1)	Once a Month (2)	Month (3)	Week (4)	Week (5)	Every Day (6)
17a. I feel I look after certain clients impersonally, as if they are objects.							
17b. I feel tired when I get up in the morning and have to face another day at work.							
17c. I have the impression that my clients make me responsible for some of their problems.							
17d. I am at the end of my patience at the end of my work day.							
17e. I really don't care about what happens to some of my clients.							
17f. I have become more insensitive to people since I've started working in labor and delivery.							
17g. I'm afraid that working in labor and delivery is making me uncaring.							

Section III: Standard of Care and Improvement

18. How would you describe the state of care in your facility?<br><br>*Read responses aloud, and select one response*	__ The facility provides excellent care with little to improve<br>__ The facility provides good care with a few areas to improve<br>__ The facility provides satisfactory care with several areas to Improve<br>__ The facility does not provide good care and could improve in many areas	**1**<br>**2**<br>**3**<br><br>**4**
19. The care provided by my colleagues is:<br><br>*Read responses aloud*	__ Poor<br>__ Fair<br>__ Good<br>__ Very Good<br>__ Excellent	**1**<br>**2**<br>**3**<br>**4**<br>**5**
20. The way my colleagues treat their clients during labor and delivery is:<br><br>*Read responses aloud*	__ Totally unacceptable<br>__ Unacceptable<br>__ Neutral<br>__ Acceptable<br>__ Perfectly Acceptable	**1**<br>**2**<br>**3**<br>**4**<br>**5**
21. How satisfied are clients with the care they receive during labor and delivery?<br><br>*Read responses aloud*	__ Very dissatisfied<br>__ Moderately dissatisfied<br>__ Neither satisfied nor dissatisfied<br>__ Moderately satisfied<br>__ Very satisfied	**1**<br>**2**<br>**3**<br>**4**<br>**5**

22. How important is client satisfaction to your work?<br><br>*Read responses aloud*	__ Not at all important<br>__ Slightly Important<br>__ Neutral<br>__ Moderately Important<br>__ Extremely Important	**1**<br>**2**<br>**3**<br>**4**<br>**5**
23. Please indicate your level of satisfaction for the following prompts.<br><br>The choices are very dissatisfied, moderately dissatisfied, neither satisfied nor dissatisfied, moderately satisfied or very satisfied.	a. Very dissatisfied<br>b. Moderately dissatisfied<br>c. Neither satisfied nor dissatisfied<br>d. Moderately satisfied<br>e. Very satisfied	
23a.	___Your overall satisfaction in your job	
23b.	___ Your ability to make decisions and control conditions in your ward	
23c.	___ Your sense of meaning and purpose in your work	
23d.	___ Your ability to improve satisfaction and care of clients	
24. Please indicate to what extent you and your colleagues can improve a clients' experience during delivery?<br><br>*Read each response aloud and select one response.*	__ There is nothing I can do to improve client experience<br>__ There is not much I can do to improve client experience<br>__ There are some things I can do to improve experience for clients<br>__ There is much I can do to improve client experience	**1**<br>**2**<br>**3**<br><br>**4**
25. How interested are you in improving the care in your facility?	__ Not at all interested<br>__ Slightly Interested<br>__ Moderately Interested<br>__ Very interested<br>__ Extremely Interested	**1**<br>**2**<br>**3**<br>**4**<br>**5**

Thank you for your time.

Health Facility ID: _ _
Date of Interview: _ _ / _ _ / _ _

Health Facility In-Charge Survey Instrument

To begin, I'd like to learn a bit more about you and your work at the health facility.

1. Tell me about your role at the health facility. How long have you served in this role?

I'd like to ask you some questions about the experience of the health facility related to the Respectful Maternity Care project implemented in collaboration with SM360+. As part of this project we have introduced five different solutions to the health facility; the BETTER pain management toolkit, the Feedback box, the Provider-Client Promise and the Facility Fresh Start funds, as well as the reflection workshop hosted earlier.

2. Have providers incorporated the new tools into their work? If so, how?

3. Providers in your facility participated in a 'Reflection Workshop' to discuss provider experiences and ideas.
    a. How did providers respond to the workshop?
    b. Have you seen providers change their behavior after the workshop?
        i. Has the workshop changed how providers see their role? How?
        ii. Has the workshop changed how providers interact with clients? How?
    c. Could you tell me about any new ideas or suggestions for improving care that came out of the discussion?

I'd like to talk in more detail about the implementation of the specific tools.

4. Has your facility incorporated the BETTER pain management toolkit into existing practices? If so, how did you incorporate it into existing practices?
    a. Do you see providers using components of the toolkit? If so, what tools do they use and how and when do they use the tools?

5. Do you see providers using the manual?
    a. How do they use it?
    b. At what moment do they use it?
    c. Do you know where you can find the manual in the facility? If so, where?

6. What do providers say about the manual?

7. What do providers find most challenging about using the manual?
    a. Have you addressed these challenges? If so, how?
    b. Are there ways we can make the manual more useful to providers?

8. Do you see providers using BETTER pain management techniques?
    i. If so, which ones?
    ii. How often do they use them?
    iii. At what moments do they use a pain management technique?
    iv. Are there techniques you see providers using more than others? If so, which techniques and what do they like about them?
    v. Has the use of the pain management techniques changed your interactions with clients during labor and delivery?

9. What challenges do providers have in using the techniques?
    a. Are certain techniques more challenging than others?
    b. Have you or other providers addressed these challenges? If so, how?
    c. Are there ways we can make the techniques more useful to providers?

Health Facility ID: _ _
Date of Interview: _ _ / _ _ / _ _

10. Are clients aware of the BETTER pain management techniques?
    a. Do clients request pain management support?
    b. Do clients look at the BETTER pain management posters?
    c. What do they say about the pain management techniques?
    d. Do you think these techniques have changed the client experience? If so, how?

11. Have you seen providers using the BETTER partograph guide? If yes,
    a. How are providers using the guides? At what moment do they use it?
    b. Has the BETTER partograph guide changed how providers interact with clients? If so, how?

12. What do providers say about the BETTER partograph?

13. Do providers experience challenges in using the partograph guide?
    a. Have you or other providers addressed these challenges in any way? How?
    b. Are there ways we can make the partograph guide more useful to providers?

14. Are providers using the Provider-Client Promise? How often do they use it? How are providers using the Provider-Client Promise?
    a. When do they administer the promise?
    b. Are there situations when they don't use the promise? What are these situations?
    c. Have there been any challenges in implementing the promise?
    d. Have you or other providers addressed these challenges? If so, how?
    e. Are there ways we can make the Promise more useful to the provider and the client?

15. What do providers say about the Promise?
    a. Has the Promise changed how providers interact with clients? If so, how?

16. How have clients responded to the Provider-Client Promise?
    a. Has the Promise changed how clients interact with providers? If so, how?

17. Have you seen a feedback box in your facility? If yes,
    a) Do you know what instructions providers give clients on how to use the box? What are they?
    b) When do they give these instructions?
    c) What is challenging about the feedback box? Have you addressed these challenges in any way? How?
    d) What are the benefits of using the feedback box?
    e) Over the past two weeks, do you know what proportion of your clients have been offered a feedback token before they leave?
    f) Were there times that a provider did not offer a token to a patient? What happened?
    g) What are some other reasons a provider might not provide a feedback token to the client?
    h) Are there ways we can make the feedback box more useful to the provider and patient?

18. What do providers say about the Feedback Box?

19. What do clients say about the Feedback Box?

20. Are you using the results of the feedback? If so, how?
    a. Who opens the feedback box to see the tokens?
    b. Do you and your colleagues discuss the feedback you receive? How often and with whom? At what moments do you discuss?
    c. How do you address issues that may arise from the feedback received?

21. Which of these tools are more helpful than the others? For what reasons?

a. Are some tools more likely to be used by providers? If so, which ones and why?

22. Did you receive "fresh start" funds for your facility? How have you used these funds? When did you use them?
    a) Can you describe these changes?
    b) How did you decide on making these changes?
    c) What has been the result of these changes?

23. Is there anything else you'd like to share with us?

Thank you for your time!

Health Facility Name _______________
Health Facility ID _ _
Date __ __/ __ __/ __ __ (DD/MM/YY)
Pre/Post Survey ____________ (1= Pre, 2=Post)

Client Endline Survey Instrument

Section I: Provision of care

Today I'd like to talk to you about the care you received during your recent delivery at the health facility.
To start, let me first learn a little about you.

Question	Answer choices	Data Entry
1. How old are you?	__ __ years	
2. How many children do you have?	*Select one*<br>__ 1<br>__ 2<br>__ 3<br>__ 4+	 **1**<br>**2**<br>**3**<br>**4**
3. What is your marital status?	*Select one*<br>__ Never Married<br>__ Living together<br>__ Married<br>__ Divorced/ Separated<br>__ Widowed	**1**<br>**2**<br>**3**<br>**4**<br>**5**
4. At what facility did you deliver your youngest baby?	__ Kasenengwa Zonal RHC<br>__ Kamlaza RHC<br>__ Mkanda Zonal RHC<br>__ Chinunda RHC<br>__ Chiparamba Zonal RHC<br>__ Madzimawe<br>__ Kapata Zonal RHC<br>__ Namseche RHC<br>__ Chikando Zonal RHC<br>__ Champhande RHC	**1**<br>**2**<br>**3**<br>**4**<br>**5**<br>**6**<br>**7**<br>**8**<br>**9**<br>**10**
5. How old is your youngest baby?	________ months	
6. Who was the main person that delivered your baby?	*Read aloud and select one response*<br>__ Doctor<br>__ Nurse<br>__ Midwife<br>__ Other __________<br>__ Do not know	 **1**<br>**2**<br>**3**<br>**4**<br>**5**
7. Do you know the name of the provider?	__ YES<br>__ NO<br><br>If no, skip to question 8	**1**<br>**0**
7a. If so, can you please provide the name		

8. How did you feel about the way the provider(s) treated you during labor and delivery?	*Read prompt aloud and select one*<br><br>__ Very satisfied<br>__ Somewhat satisfied<br>__ Somewhat dissatisfied<br>__ Very dissatisfied	 <br>**4**<br>**3**<br>**2**<br>**1**
9. Which of the following words describes the provider who attended to you?<br><br>9a.<br>9b.<br>9c.<br>9d.<br>9e.<br>9f.<br>9g.	*Read all responses below and select all that apply*<br><br><br>__ Kind/Friendly<br>__ Skilled<br>__ Patient<br>__ Rude/Harsh<br>__ Impatient<br>__ Collaborative<br>__ Violent	**YES=1**<br>**NO=0**

Each of the questions below relate to your expectations <u>prior</u> to going to the facility for your most recent delivery.

Please respond whether you agree or disagree with each statement

10. I expected my provider to provide good care	__ Agree<br>__ Disagree	**1**<br>**0**
11. I expected my provider to yell or scold at me during labor and delivery	__ Agree<br>__ Disagree	**1**<br>**0**
12. I expected to have privacy during labor or delivery	__ Agree<br>__ Disagree	**1**<br>**0**
13. I expected that the provider might use insults, intimidations, threats or coercion	__ Agree<br>__ Disagree	**1**<br>**0**
14. I expected that I would have a safe delivery	__ Agree<br>__ Disagree	**1**<br>**0**
15. I expected my provider to help me manage my pain	__ Agree<br>__ Disagree	**1**<br>**0**

16. At any point during your delivery did the provider seem:<br><br>16a.<br>16b.<br>16c<br>16d.	*Read prompt aloud and select all that apply*<br><br>__ Angry<br>__ Stressed<br>__ Nervous<br>__ Disgusted	**YES=1**<br>**NO=O**
17. How painful was your delivery?	*Read responses aloud and select one*<br>__ Unbearable pain<br>__ Severe pain<br>__ Moderate pain<br>__ Slight pain	 **1**<br>**2**<br>**3**<br>**4**

	__ No pain	**5**
18. Did you ask the provider to help you when you were feeling pain?	__ YES<br>__ NO	**1**<br>**0**
19. Did the provider do anything to make you feel better or alleviate the pain?	__ YES<br>__ NO<br>If no, skip to question 21	**1**<br>**0**
20. If yes, what did they do?<br><br>20a.<br>20b.<br><br>20c.<br>20d.<br><br>20e.<br><br>20f.<br>20g.<br>20h.	*Read options aloud and select all that apply*<br><br>__ Breathing exercises<br>__ Helped you think distracting or positive thoughts<br>__ Encouraged you<br>__ Helped you change position/walk around<br>__ Rubbed/massaged you with hand or ball<br>__ Chatted with you<br>__ Gave medication<br>__ Other<br>Please specify: ________________	**YES=1**<br>**NO=O**
21. How important do you think your satisfaction was to the provider?	*Read responses aloud then select one*<br><br>__ Not important<br>__ Of minimal importance<br>__ Important<br>__ Very important	 <br>**1**<br>**2**<br>**3**<br>**4**

Each of the statements below relate to your experience <u>during</u> labor and delivery during your most recent delivery.

Please respond whether you agree or disagree

22. My provider treated me well during my delivery	__ Agree<br>__ Disagree	**1**<br>**0**
23. I trusted my provider to manage my delivery	__ Agree<br>__ Disagree	**1**<br>**0**
24. My provider cared about me and how I was feeling.	__ Agree<br>__ Disagree	**1**<br>**0**
25. I accepted that my provider might need to yell at me or scold me during labor and delivery	__ Agree<br>__ Disagree	**1**<br>**0**
26. My provider was available and listened to me during labor and delivery	__ Agree<br>__ Disagree	**1**<br>**0**

27. Did you experience any of the following during childbirth when at the facility?<br>27a.<br>27b.	*Read each prompt aloud and have client select all that apply*<br><br>__ Provider made you feel uncomfortable<br>__ Provider shouted/scolded or said something	*Enter*<br>*1=YES*<br>*0= NO*

27c.<br>27d.<br><br>27e.<br><br>27f.<br><br>27g.<br>27h.<br>27i.	harsh or unkind<br>__ Provider threatened to withhold treatment<br>__ Provider made threatening, negative or discouraging comments<br>__ Your body was seen by others, or your privacy Was violated in another way<br>__ Provider ignored or abandoned you when in need<br>__ You delivered alone<br>__ You had a surgery done without consenting to it<br>__ Provider hit, slapped, pushed, pinched, or otherwise beat you	
28. If you were dissatisfied with the care you received, or if something were to happen during your delivery to make you feel dissatisfied, would you feel comfortable making a complaint?	__ YES<br>__ NO	**1**<br>**0**
29. How would you file a complaint, if you decided to?	*Read response aloud and select best response*<br><br>__ Notify facility staff<br>__ Tell my provider directly<br>__ Tell the hospital in-charge<br>__ Other, please specify ________________	 <br>**1**<br>**2**<br>**3**<br>**4**

Section II: Satisfaction with Service

30. Which factors did you consider when choosing this facility for your last delivery?<br><br>30a.<br>30b.<br>30c.<br>30d.<br>30e.<br><br>30f.<br>30g.<br>30h.<br>30i.<br>30j.<br>30k.<br>30l.<br>30m.	*Read the question aloud, and let women respond open-ended. Mark all answers that women say aloud.*<br><br>__ Closest facility<br>__ Facility provides good clinical care<br>__ Providers treat clients well<br>__ Same facility used for ANC<br>__ To get services not available elsewhere<br>__ Affordable services<br>__ Recommended by relative/friend<br>__ Avoid congestion in other facilities<br>__ Referred from another facility<br>__ Only option<br>__ Facility offers specialized services<br>__ Decision made by spouse<br>__ Other<br>Please Specify ____________	*Enter*<br>*1=YES*<br>*0= NO*
31. How would you rate the care you received for this delivery?	*Read response options aloud and select one response*<br>__ Excellent	 <br>**5**

	___ Very good<br>___ Good<br>___ Fair<br>___ Poor	**4**<br>**3**<br>**2**<br>**1**
32. Would you recommend this facility to a friend or relative for delivery?	___ YES<br>___ NO<br>___ UNDECIDED	**2**<br>**1**<br>**0**

33. How would you rate the importance of the following in deciding where to have a future delivery?

The response options are not at all important, slightly important, neutral, moderately important or extremely important

	Not at all important (1)	Slightly Important (2)	Neutral (3)	Moderately Important (4)	Extremely Important (5)
33a. Location of the health facility					
33b. The clinical knowledge of the provider					
33c. How the provider treats you					

Section IV: Other and Future health services

34. Have you returned to any health facility since giving birth?	___Yes<br>___No<br>**If yes, continue to question 35**<br>**If no, skip to question 39**	**Yes=1**<br>**No=0**
If yes, please tell me which of these services you returned for and whether you returned to this facility or another one.<br><br>*Read each service aloud*		**1= Yes**<br>**0= No**
35a	___ Postnatal check up	**1**<br>**0**
35b.	___ Returned to this facility<br>___ Returned to other facility	**1**<br>**0**
36a.	___ Immunizations	**1**<br>**0**
36b.	___ Returned to this facility<br>___ Returned to other facility	**1**<br>**0**
37a.	___ Family planning	**1**<br>**0**
37b.	___ Returned to this facility<br>___ Returned to other facility	**1**<br>**0**
38.a	___ Other reason<br>Please Specify ________________________	**1**<br>**0**
38b.	___ Returned to this facility<br>___ Returned to other facility<br><br>**Skip to question 40**	**1**<br>**0**

39. If no, what are your reasons for not returning to the health facility	*Do not read out loud, instead listen and note the appropriate response*<br><br>__ Have not sought health services<br>__ Did not like the care I received during my last visit<br>__ Received care/services through community health workers<br>__ Other<br>*Please specify* ______________	 <br>**1**<br>**2**<br><br>**3**<br><br>**4**

43. Would you return to the same facility for a future delivery?	__ YES<br>__ NO<br>__ UNDECIDED	**2**<br>**1**<br>**0**
44. Why would or would you not return to the facility for a future delivery?<br><br>41a.<br>41b.<br>41c.<br>41d.<br>41e.<br>41f.<br>41g	*Do no read prompts, but select all responses related to client answers*<br><br>__ Location of facility<br>__ Quality of clinical care<br>__ How Providers treat clients<br>__ Services available<br>__ Cost of services<br>__ Only option<br>__ Other<br>Please Specify____________________	*Enter*<br>*1=YES*<br>*0= NO*
45. The next several questions are hypothetical. If a friend or relative was considering delivery at the health facility, what would you tell them to expect?<br><br>Please state if you agree or disagree with what your friend or relative should expect.		**Agree=1**<br>**Disagree =0**
42a. They should expect the provider to welcome them	__ Agree<br>__ Disagree	
42b. They should expect that the provider might use physical force, such as slapping or hitting	__ Agree<br>__ Disagree	
42c. They should expect that a provider might use insults or threats	__ Agree<br>__ Disagree	
42d. They should not expect to have privacy during labor and delivery	__ Agree<br>__ Disagree	
42e. They should expect that the provider may yell or be harsh with them	__ Agree<br>__ Disagree	

Section IV: Use of designs

For implementation sites only

46. Did a provider administer the "Provider-client promise" when you first arrived at the labor and delivery ward?<br>(*If client does not recognize the provider-client promise, describe the*	__YES<br>__NO	1=YES<br>0=NO

document. Did they read a document that stated the provider and client's responsibilities during labor and delivery and had you sign it?)		
47. Did a provider give you a token when you departed the labor and delivery ward?	__YES<br>__NO	
48. Did the provider tell you what to do with the token?	__YES<br>__NO<br><br>If no, continue to question 47.	
49. If yes, what instructions did they provide?	__ Provider gave token and told to drop into whichever best represents her experience<br><br>__ Provider gave patient coin and told her which slot to insert the coin.<br><br>__ Other<br>Please specify_________________	
50. Did you see a "BETTER" pain management techniques poster in the health facility?	__YES<br>__NO<br><br>If no, skip to question 50.	
51. If yes, where was the poster located?	*Read all responses aloud and select all that apply*<br><br>__ in the delivery room<br>__ in the ANC waiting room<br>__ in the waiting room<br>__ others<br>Please specify ________________	

Section V: Qualitative questions

I'd like to ask you a few other questions about your recent experience delivering at the health facility.

(To ask all clients)

1. What were your expectations when you went to the facility for your most recent delivery? How did you develop those expectations?

2. Were your expectations met?
    a. If not, why?
    b. If yes, what did they provider do to meet those expectations?
    c. Could the provider have done something differently to change your experience?

3. Did you like the facility where you delivered? What did you dislike? What did you like?

4. Was there anything that went particularly well during delivery? If so, can you please describe it.
    a. Was there anything your provider did that you appreciated?

5. Was there anything that did not go well during delivery?
    a. Did the provider do anything that upset you?

6. If this was not your first delivery, were there any differences between this delivery and your last? If so, can you please describe those differences?
    a. Why do you think it was different?

7. How did you feel when you were given your baby for the first time?

8. What did you do once you were given your baby?
    a. How did your feelings for your baby develop after delivery?

9. When did you first experience positive feelings and love towards them?
    a. What do you and your baby do together (cuddling, talking, playing?)
    Potential probes: How do you feel when your baby cries? When your baby wakes you at night?

(To ask clients who delivered in implementation facilities)

10. (If promise was conducted) When the provider conducted the provider-client promise, what did you think of this promise? *The promise was a series of agreements that the provider read outload that stated both the provider and your responsibilities during delivery.*
    a. How did you feel?
    b. Did the provider maintain their promise?
    c. Did the provider behave differently because of the promise? If so, how?

11. (If given token) When you were given the token, what did you think?
    a. How did you feel about being given a token?
    b. How did you feel about providing feedback?
    c. How did you envision the feedback would be used?

(To ask all clients)

12. Is there anything else about your labor and delivery experience that you would like to share?

Name of Health Facility: ____
Health Facility Code: _ _
Date Conducted: _ _/_ _/_ _ (DD/MM/YY)
Survey ID: P _ _ _ _

Endline Provider Survey Instrument

Section 1: Provider Role

To start, I'd like to learn more about your role at the health facility as it relates to labor and delivery.

Question	Answer choices	Data Entry
1. How old are you?	|__| __| years	
2. What is your gender?	__ Male<br>__ Female	**0**<br>**1**
3. What is your title?	__ Nurse<br>__ Midwife<br>__ Doctor<br>__ Other, please specify ____________	**1**<br>**2**<br>**3**<br>**4**
**4.** How many years of experience do you have attending deliveries?	|__| __| years	
5. How many deliveries did you attend over the last two weeks?	*Select one*<br>__ 0<br>__ 1-4<br>__ 5-9<br>__ 10-14<br>__ 15-19<br>__ 20-29<br>__ 30-39<br>__ ≥40	 **1**<br>**2**<br>**3**<br>**4**<br>**5**<br>**6**<br>**7**<br>**8**
6. What are the three most important things a provider does during delivery?<br><br>I will read the choices below and please select 3.<br><br>*Read responses below and then note the three most important below under 6a-6c.*<br>*For data entry enter the corresponding number in the data entry fields for the three response*		
	Deliver a healthy baby	**1**
	Encourage and support the client during delivery	**2**
	Handle any complications that arise	**3**
	Ensure proper documentation	**4**
	Monitor the delivery	**5**
	Help the client to manage pain	**6**
	Ensure the client follows instructions	**7**

	Other	**8**
	Please specify : ___________________________	
6a.<br>6b.<br>6c.	Response 1 __<br>Response 2 __<br>Response 3 __	

7. Which of the following statements do you most agree with?

I will read the three responses below, and then please select the one you agree with most.

Read the prompts below, select one response.

	The thing that matters most is that the baby and mother are healthy	**1**
	It matters that the baby and mother are healthy and the mother is satisfied with the care she received	**2**
	It only matters that the mother is satisfied with the care she received.	**3**

8. How important is it to you to provide support and encouragement during labor and delivery?	*Read prompts aloud and select one response*<br><br>___ Not at all important<br>___ Slightly Important<br>___ Moderately Important<br>___ Very Important<br>___ Extremely Important	 <br>**1**<br>**2**<br>**3**<br>**4**<br>**5**
9. If a client is experiencing pain during labor, is there something that you do to help her manage the pain?	___ YES<br>___ NO<br><br>If no, proceed to question 10.	**1**<br>**0**
9a. What techniques can be used to manage pain?<br><br>9a1.<br>9a2.<br>9a3.<br>9a4.<br>9a5.<br>9a6.<br>9a7.<br>9a8.<br>9a9.	*Do not read. Mark the corresponding answer, and select all that apply*<br><br>___ Breathing exercises<br>___ Encourage with positive or distracting thoughts<br>___ Encouragement<br>___ Change position/walk around<br>___ Rub/massage with hand or ball<br>___ Chat with her<br>___ Gave medication<br>___ Nothing<br>___ Other<br>*Please explain-*__________________	**1= Yes**<br>**0=No**
9b. How frequently do you use the techniques?	*Read all responses aloud and select one response*<br><br>___ During every delivery<br>___ During most deliveries<br>___ During some deliveries<br>___ Rarely<br>___ Never	 <br>**5**<br>**4**<br>**3**<br>**2**<br>**1**

9c. In the last 30 days, has a client requested pain management support during labor and delivery?	__ YES<br>__ NO<br><br>If no, proceed to question 10	**1**<br>**0**
9d. If yes, did you provide pain management support?	__YES<br>__ NO<br><br>If no, proceed to question 10	**1**<br>**0**
9e. What techniques did you use?<br><br>9e1.<br>9e2.<br>9e3.<br>9e4.<br>9e5.<br>9e6.<br>9e7.<br>9e8.<br>9e9.	*Do not read. Mark the corresponding answer, and select all that apply*<br><br>__ Breathing exercises<br>__ Encourage with positive or distracting thoughts<br>__ Encouragement<br>__ Change position/walk around<br>__ Rub/massage with hand or ball<br>__ Chat with her<br>__ Gave medication<br>__ Nothing<br>__ Other<br>*Please explain-*__________________	**1= Yes**<br>**0=No**

Section II: Provision of Care

10. Please indicate your level of agreement with the following prompts.
The choices are strongly disagree, disagree, neither agree or disagree, agree, and strongly agree

Read each prompt aloud.

	Strongly Disagree (1)	Disagree (2)	Neither Agree or Disagree (3)	Agree (4)	Strongly Agree (5)
10a. When giving birth, clients are often cooperative					
10b. Clients who have a low level of education are less likely than other clients to follow instructions					
10c. It is sometimes necessary to scold a client during labor and delivery					
10d. Clients often do not want to listen to providers during labor and delivery					

10e. Yelling at a client during labor and delivery ensures that she cooperates during procedures					
10f. Clients in labor or in delivery are deserving of kindness					

11. Have you witnessed the following actions in the labor and delivery ward?

For each action you will be asked whether you have ever witnessed the action, if you have witnessed the action in the last 2 weeks, or you have never witnessed the action

	Never (0)	Yes, within the past 2 weeks (1)	Yes, ever in the past (2)
11a. Service providers use physical force with laboring clients (such as slapping or hitting)			
11b. Service providers use insults, intimidation, threats or coercion towards clients or their companions			
11c. Service providers show disrespect to clients based on any specific attribute (such as marital status, HIV status, economic status, age)			
11d. Service providers scold a client for her behavior or yell at her			

12. In your own personal capacity have you ever done anything that you consider to be disrespect or abuse toward a client in childbirth?	__ YES<br>__ NO	**1=YES**<br>**0=NO**
13. Please evaluate this statement: Providers in my facility believe that yelling and scolding a patient is:	*Read responses aloud and select one response*<br><br>__ Is never acceptable<br>__ Isn't acceptable but sometimes is necessary<br>__ Is acceptable when it is necessary<br>__ Always acceptable	**1**<br>**2**<br>**3**<br>**4**

14. The next questions ask you about your feelings and thoughts during the last month. In each case, you will be asked to indicate how often you felt or thought a certain way.

The choices are never, almost never, sometimes, fairly often, and very often

	**Never (0)**	**Almost Never (1)**	**Sometimes (2)**	**Fairly Often (3)**	**Very Often (4)**

29

14a. In the last month, how often have you felt that you were unable to control outcomes for clients in the ward?					
14b. In the last month, how often have you felt nervous and "stressed" when attending to a delivery?					
14c. In the last month, how often have you felt confident about your ability to handle problems in the delivery ward?					
14d. In the last month, how often have you been able to control irritations in your life?					
14e. In the last month, how often have you been angered about something that happened during a delivery because of things that were outside of your control?					

15. Please indicate your level of agreement with the following prompts.

The choices are strongly disagree, disagree, neither agree or disagree, agree, and strongly agree

	Strongly disagree (1)	Disagree (2)	Neither agree or disagree (3)	Agree (4)	Strongly agree (5)
15a. I try to imagine myself in my clients' life when providing care to them					
15b. I try to understand what is going on in my clients' minds by paying attention to their nonverbal cues and body language					
15c. I believe that empathy is an important therapeutic factor in medical treatment					
15d. My clients feel better when I understand their feelings					
15e. I try not to pay attention to my clients' emotions in interviewing and history taking					
15f. It is difficult for me to view things from my clients' perspectives					

16. Please indicate your level of agreement with the following prompts.

The choices are strongly disagree, disagree, neither agree or disagree, agree, and strongly agree

	Strongly Disagree (1)	Disagree (2)	Neither agree or disagree (3)	Agree (4)	Strongly Agree (5)
16a. I prefer to wash my hands pretty soon after shaking someone's hand					
16b. It does not make me anxious to be around sick people					
16c. My hands do not feel dirty after touching money					
16d. The more educated a person is, the better their hygiene is likely to be					
16e. I feel ill or disgusted when I see blood or other bodily fluids on my clothing or hands					
16f. It is wrong that unmarried girls have sex and get pregnant					

17. For each question, please tell me how often you may experience the feelings described.

The choices are never, a few times per year, once a month, a few times per month, once a week, a few times per week, and every day

	Never (0)	A Few Times per Year (1)	Once a Month (2)	A Few Times per Month (3)	Once a Week (4)	A Few Times per Week (5)	Every Day (6)
17a. I feel I look after certain clients impersonally, as if they are objects.							
17b. I feel tired when I get up in the morning and have to face another day at work.							
17c. I have the impression that my clients make me responsible for some of their problems.							
17d. I am at the end of my patience at the end of my work day.							
17e. I really don't care about what happens to some of my clients.							
17f. I have become more insensitive to people since I've started working in labor and delivery.							
17g. I'm afraid that working in labor and delivery is making me uncaring.							

31

Section III: Standard of Care and Improvement

18. How would you describe the state of care in your facility?<br><br>*Read responses aloud, and select one response*	__ The facility provides excellent care with little to improve<br>__ The facility provides good care with a few areas to improve<br>__ The facility provides satisfactory care with several areas to Improve<br>__ The facility does not provide good care and could improve in many areas	**1**<br>**2**<br>**3**<br><br>**4**
19. The care provided by my colleagues is:<br><br>*Read responses aloud*	__ Poor<br>__ Fair<br>__ Good<br>__ Very Good<br>__ Excellent	**1**<br>**2**<br>**3**<br>**4**<br>**5**
20. The way my colleagues treat their clients during labor and delivery is:<br><br>*Read responses aloud*	__ Totally unacceptable<br>__ Unacceptable<br>__ Neutral<br>__ Acceptable<br>__ Perfectly Acceptable	**1**<br>**2**<br>**3**<br>**4**<br>**5**
21. How satisfied are clients with the care they receive during labor and delivery?<br><br>*Read responses aloud*	__ Very dissatisfied<br>__ Moderately dissatisfied<br>__ Neither satisfied nor dissatisfied<br>__ Moderately satisfied<br>__ Very satisfied	**1**<br>**2**<br>**3**<br>**4**<br>**5**
22. How important is client satisfaction to your work?<br><br>*Read responses aloud*	__ Not at all important<br>__ Slightly Important<br>__ Neutral<br>__ Moderately Important<br>__ Extremely Important	**1**<br>**2**<br>**3**<br>**4**<br>**5**
23. Please indicate your level of satisfaction for the following prompts.<br><br>The choices are very dissatisfied, moderately dissatisfied, neither satisfied nor dissatisfied, moderately satisfied or very satisfied.	a. Very dissatisfied<br>b. Moderately dissatisfied<br>c. Neither satisfied nor dissatisfied<br>d. Moderately satisfied<br>e. Very satisfied	
23a.	___Your overall satisfaction in your job	
23b.	____ Your ability to make decisions and control conditions in your ward	
23c.	____ Your sense of meaning and purpose in your work	
23d.	____ Your ability to improve satisfaction and care of clients	

24. Please indicate to what extent you and your colleagues can improve a clients' experience during delivery?<br><br>*Read each response aloud and select one response.*	__ There is nothing I can do to improve client experience<br>__ There is not much I can do to improve client experience<br>__ There are some things I can do to improve experience for clients<br>__ There is much I can do to improve client experience	**1**<br>**2**<br>**3**<br><br>**4**
25. How interested are you in improving the care in your facility?	__ Not at all interested<br>__ Slightly Interested<br>__ Moderately Interested<br>__ Very interested<br>__ Extremely Interested<br><br>If Not at all interested, skip to question 27.	**1**<br>**2**<br>**3**<br>**4**<br>**5**

Section IV: Qualitative Questions

26. You said that you are [*read answer to 25 here*] interested in improving the care in your facility.

a. What are some of the things that you would like to see improved? Why?
b. How would you go about improving these things?
c. Can you give me an example of a recent improvement that was made to your facility?
d. How did this affect labor and delivery and clients' experiences?
e. Were there any other recent improvements made to your facility? What?

27. Earlier on, we discussed clients' satisfaction with the care they receive during labor and delivery.

- ☐ What are some ways you can tell if a client is satisfied? If they are unsatisfied?
- ☐ What are some things that make clients satisfied? Unsatisfied?
- ☐ Do you ever discuss client satisfaction with your colleagues? What do you talk about?
- ☐ Has client satisfaction changed in any way recently?
  - **i.** Did it improve or get worse?
  - **ii.** What happened to lead to this change?

28. What are some things you can do to make clients more satisfied and to improve their experiences during labor and delivery?

Refer to question 10a.

29. You noted that you [*read answer to 10a here*] that clients are often cooperative during labor and delivery. Are there any techniques you use can use to make clients more cooperative?

- ☐ What are these techniques?
- ☐ When would you use them?

33

30. We also discussed pain management techniques that you can use to help clients during labor and delivery and you mentioned a few to me.
   - ☐ How do you decide when you use a technique?
   - ☐ How do you decide which techniques to use?
   - ☐ Where did you learn about these techniques?
   - ☐ How do clients respond to the different techniques?

31. Thinking back to actions you may have witnessed other providers do in the labor ward - such as using physical force, intimidation and scolding – how do you typically notice these actions taking place?
   - ☐ Where are you and what are you doing when you notice?
   - ☐ What do you think when you see these actions happening?
   - ☐ Do you do anything in the moment or afterwards? What do you do?

Refer to question 12.

32. In your own personal capacity, you said that you [*HAVE or HAVE NOT*] done anything you consider to be disrespectful towards a client during childbirth.
   - ☐ What are some reasons you answered the question in this way?
   - ☐ How did you recognize what you were doing was respectful/disrespectful?
   - ☐ Can you give me an example of some of the respectful/disrespectful actions you've done?
   - ☐ How do these actions make you feel?

Section V: Design Implementation (for implementation facilities only)

33. Have you seen any pain management posters in your facility? If yes,
   a. Where are the posters located?
   b. What does the poster describe?
   c. Do you use the poster? (*If not, probe why they have not used the tool*)
      i. How do you use it?
      ii. At what moment do you use it?
      iii. How often do you use it?
      iv. Are there any benefits of using the it? What are they?
      v. Has it changed your interactions with clients?
      vi. Has a client ever said anything to you about the poster? What did she say?
      vii. Is anything challenging about using it?
      viii. Did you address these challenges in any way? How?
   d. Are there ways we can make the poster more useful to providers?
   e. Do you see other providers using the poster? What do they think about it?

34. Have you seen the BETTER pain management manual? If yes,
   a. Do you know where you can find the manual in the facility? If so, where?
   b. Have you used the manual? (*If not, probe why they have not used the tool*)
      i. How do you use it?

ii. At what moment do you use it?
iii. How often do you use it?
iv. Has it changed your interactions with clients?
v. Is anything challenging about using it?
vi. Did you address these challenges in any way? How?

c. Are there ways we can make the manual more useful to providers?
d. Do you see other providers using the manual? What do they think about it?

35. Have you seen the BETTER partograph guide? If yes,

c. Do you know where you can find the BETTER partograph guide in the facility? If so, where?
d. Have you used the BETTER partograph guide? (*If not, probe why they have not used the tool)*
  i. How do you use it?
  ii. At what moment do you use it?
  iii. Has it changed your interactions with clients?
  iv. How often do you use it?
  v. Is anything challenging about using it?
  vi. Did you address these challenges in any way? How?
e. Are there ways we can make the BETTER partograph guide more useful to providers?
f. Do you see other providers using the BETTER partograph? What do they think about it?

36. Have you seen the Provider-Client Promise document? If yes,

a. Do you know where you can find the Provider-Client Promise document in the facility? If so, where?
b. Have you used the Provider-Client Promise? (*If not, probe why they have not used the tool)*
  i. How do you use it?
  ii. At what moment do you use it?
  iii. How often do you use it?
  iv. Is anything challenging about using it?
  v. Did you address these challenges in any way? How?
  vi. Are there any benefits of using it? What are they?
c. What happens after you complete the Promise document?
d. What do patients think about the Promise document?
e. Are there ways we can make the Promise more useful to the provider and the client?
f. Do you see other providers using the Promise? What do they think about it?

37. Have you seen a feedback box in your facility? If yes,

a. Do you know where you can find the feedback box in the facility? If so, where?
b. Have you used the feedback box? (*If not, probe why they have not used the tool)*
  i. How have you used it?
  ii. What instructions do you provide to patients?
  iii. At what moment do you use it?
  iv. How often do you use it?
  v. Is anything challenging about it?
  vi. Did you address these challenges in any way? How?
  vii. Are there any benefits of using it? What are they?
c. Do other providers offer patients feedback tokens?
d. What are reasons a provider would not provide a feedback token?
e. Over the past two weeks, what proportion of your patients have been offered a feedback token before they leave?

35

f. Have the results from the feedback box been shared with you? Who shared them? How was the information shared?
g. Do you or your colleagues discuss the feedback received? If so, what did you discuss?
h. Have any changes happened in the facility as a result of the feedback? What has changed? How did this happen?
i. Are there ways we can make the feedback box more useful to the provider and patient?

38. Have you participated in a 'Reflection Workshop' to discuss provider experiences and ideas? If yes,
   a. Could you tell me what you learned during the workshop?
   b. What were your favorite (and least favorite) activities and discussions?
   c. Could you tell me about any new ideas or suggestions that came out of the discussion?
   d. Have you changed the way you do your job since the workshop? What have you changed?
   e. Overall, what did you think of this workshop? Was it a good or poor use of your time?

39. Did your facility receive "Fresh Start" funds to support and improve client experience?
   a. How did you decide how the funds would be used? Who was part of these discussions?
   b. What did the facility decide to do with the funds? How was this implemented?
   c. How have clients responded to the changes? How have providers responded?

Thank you for your time.

36

Observation guide for Pain Management Toolkit (BETTER Poster, BETTER Pain Management Technique Manual, and BETTER Partograph Guide)

Note: the observer will need to track one client over time, from admission till delivery. Notes on one client's journey (related to the Pain Management Toolkit) should be recorded on this form. Simply observe; do not provide instruction or guidance.

Date:	Time:
Facility name:	Facility type:

	YES	NO
1. Was there a copy of the BETTER poster hung in the following locations?		
a. In the maternity ward?		
b. If in the maternity ward, next to ____ beds of ____ total beds		
c. In the antenatal room in the maternity ward?		
d. In the room where antenatal consultations are conducted in the MCH ward?		
2. Did the provider glance at any of these posters during the client's labor?		
Notes: What did the provider do after glancing at the poster?		
3. Did the client glance at the BETTER poster during labor?		
Notes: What did the client do after glancing at the poster?		
4. Was there a copy of the BETTER partograph guide displayed by the desk of the provider in the labor ward?		
5. Did the provider glance at the partograph guide during delivery?		
Notes: What did the provider do after glancing at the partograph?		
6. Do you see a copy of the BETTER Pain Management Technique Manual?		
Notes: Is it accessible during labor and delivery? Did the provider refer to the manual at all? For what?		
7. Did the client mention experiencing pain at any point during labor and delivery?		
Notes: What did she say? How did the provider respond?		
8. Did the client ask for help with her pain at any point?		
Notes: If so, when? How did the provider respond?		

37

9. Did the provider implement breathing exercises with the client?		
a. Did they encourage slow breathing during the latent phase of labor?		
b. Did they encourage fast breathing during the active phase of labor?		
Notes: How did the client seem to respond? Any other observations?		
10. Did the provider praise, encourage or reassure the client during labor?		
Notes: What did the provider say? How did the client seem to respond? Any other observations?		
11. Did the provider help or encourage the client to change position or walk around?		
Notes: How did the client seem to respond? Any other observations?		
12. Did the provider complete a progressive muscle relaxation exercise or stretching exercise with the client?		
Notes: How did the client seem to respond? Any other observations?		
13. Did the provider encourage positive or distracting thoughts with the client?		
Notes: During which stage of labor did this occur? What did the provider say? How did the client seem to respond? Any other observations?		
14. Did the provider rub the client's back?		
a. Did the provider use the massage ball?		
Notes: What prompted the massage? Where on the client's body did the provider massage? How did the client seem to respond? Any other observations?		
15. Did the provider show the client how to rub her own back using the massage ball?		
Notes:		
16. If the provider used a ball to provide a back massage for the client, did you observe it being sanitized before or afterward?		
Notes:		

38

Questions for Provider at the completion of the observation:

1. Do you have your copy of the BETTER manual with you? Where do you keep your copy of the BETTER manual? Do you refer to them during labor and delivery? If so, when?

2. Do you have your massage ball with you? Where do you usually keep it?

39

Observation guide for Feedback Box

Note: the observer will need to track one client from before discharge to until she leaves the facility. Simply observe; do not provide instruction or guidance.

Date:	Time:
Facility name:	Facility type:

	YES	NO
1. Is there a feedback box in the maternity ward?		
a. Is it located so that clients will see it as they are leaving the ward?		
b. Can providers see in which slot the clients are placing their token?		
Notes: Where in the ward (or elsewhere) is the feedback box?		
2. Upon discharge, did the provider or someone else in the facility give the client a token to put into the feedback box?		
Notes: Who gave the client a token?		
3. Did the provider instruct the client to put the token in the slot according to how she feels about the care she received?		
Notes: How did the provider explain what the patient was to do?		
4. Did the provider explain the rating system or the different emotions associated with each slot?		
Notes: How did the provider explain the slots or emotions?		
5. Did the client put her token in the feedback box?		
Notes: Did the client seemed confused or did she take a long time to put the token in the slot? Note: Please write down anything you observe about the client at this moment?		
6. Did the client herself put the token in the feedback box? If it was someone else, please state below.		
Notes: Did someone other than the client input the token? Who?		

40

7. Did the provider give the client privacy when using the feedback box? (If the provider did not attempt to see where the client put the token and was not too close to the client, respond "YES")		
Notes:		
	**YES**	**NO**
8. Did the client need any help or clarification when they reached the feedback box?		
Notes: If yes, what did she need help with? Did anyone provide help?		
9. Is the feedback box locked?		
Notes: If so, who has a key? If not, does anyone look at tokens? Who? Where? Did you see this or hear it from someone?		
10. Did any facility staff put tokens in the feedback box?		
Notes: If yes, who?		

41

Observation guide for Provider-Client Promise

Note: the observer will need to track one client over time, from admission till active labor or after the Provider-Client Promise has been completed. Simply observe; do not provide instruction or guidance.

Date:	Time:
Facility name:	Facility type:

	YES	NO
11. Was there a poster-sized copy of the Provider-Client Promise hung in the following locations?		
a. In the delivery room in the maternity ward near where providers admit clients		
b. In the antenatal room in the maternity ward		
c. In the postnatal room in the maternity ward		
Notes:		
12. Were Provider-Client Promise sheets available in the maternity ward near where providers admit clients?		
Notes:		
13. Was the Provider-Client Promise applied by the provider during admission? (upon the client's arrival)		
Notes: If not, was there a reason why not? What was it?		
14. If the promise was not applied by the provider during admission, was it applied at a later point in time?		
Notes: If so, when was it initiated?		
15. Did the provider explain the purpose of the promise?		
Notes: What did the provider explain?		
16. Did the provider read the four provider promises out loud?		
Notes: If not, which did she skip or what did she do (ex: have the client read them herself)?		
17. Did the provider explain the four provider promises as they are written?		
Notes: If not, what changes did the provider make? How did it differ to how it is written in the document?		
18. Did the provider ask the client to repeat the four client promises after her?		

42

Notes: If not, which did she skip or what did she do?		
19. Did the provider explain the four client promises as they are written?		
Notes: If not, what changes did the provider make? How did it differ to how it is written in the document?		
20. Did the client repeat the four client promises after the provider?		
Notes: If not, was there an apparent reason why not?		
21. Were there any questions or discussions about the promises, before or after the promises were said aloud?		
Notes: Who asked a question? What was it? What was discussed?		
22. Did the client make any comments or ask any questions about the promise?		
Notes: If so, what did she say?		
23. Did the provider sign or initial the Provider-Client Promise document?		
Notes: If not, why not?		
24. Did the client sign or initial the Provider-Client Promise document?		
Notes: If not, why not?		
25. If a new provider takes over caring for the client during labor and delivery, is anything done with the Promise? Does the new provider sign the Promise document?		
Notes:		
26. Did the provider add the Provider-Client Promise document to the client's paperwork file?		
Notes:		
27. If there was a companion present during the promise, how did the companion respond to this exercise? Did they say or ask anything else? *Notes:*		
28. Overall, was there anything the client or provider appeared to be confused by? *Notes:*		

43

44
